# Supplementary material for: Involving the public in epidemiological public health research: a qualitative study of public and stakeholder involvement in evaluation of a population-wide natural policy experiment
Source: BMJ Open. 2018 Apr 20;8(4):e019805. doi: 10.1136/bmjopen-2017-019805 (PMC5914713; doi:10.1136/bmjopen-2017-019805)
Supplement: Supplementary file 1 [file bmjopen-2017-019805supp001.pdf]

S1 Appendix 1: Focused Workshop standard topic guide

Public

WS-leader Expert (introduce Demetriq + results), Moderator (questions, discussion)

Dual audio recording (mp3 + smartphone)

Demetriq = Developing methodologies to reduce inequalities in the determinants of health

Schedule (times are not exact, changed flexibly according to the workshop's progress)

00: 00-00: 15/20 Introduction

Introductory Workshop - Moderator; hello-hello, my name is ... and do ... (about 5 minutes)

Purpose of the PI: audiences outside the research group will help the group achieve better research results.

The project: research process has reached the interpretation phase, key stage, but analytical results without interpretation says nothing - but: no one can know and think about everything, it also applies to scientists.

Demetriq revolves around the question: how does the development of labor market policies affect the chances of different socio-economic groups on the labor market and the risk of exclusion (which is considered to have a connection to the health inequalities between different social groups).

This is where you enter: actors with expertise with respect to the labor market were invited, who can contribute with different views than a social medicine research group has - it is you who are [name of public group] [experts], but not only you, but also

politicians, employers, unions, social insurance office, unemployment agency => to get the "full picture" to broaden the perspective, see more of the analysis results to achieve a better, more reliable interpretation

Use of mp3 player: ok if I start audio recording (capture as much info as possible)

Discussion Rules:

Remember (mp3 player):

- To talk without interruption + to talk one at a time
- To describe the piece of data you refer to with words
- Questions always welcome

Consent:

voluntary – stop at anytime

anonymous - no accounting at the individual level or by name (in the published articles and reports)

confidential - datasets only available to the research team

- Consent in presentation round

Presentation Round: name, position in the organization, consent (identifiable voice on "tape")

Introduction Demetriq - Expert (about 10-15 minutes)

Finish with questions

00: 20 to 00:50: Discussion part I: Employment

<u>1a) presented results (20min): Empty timeline (1) + Employment Charts Sweden (2)</u>

- If you think of the employment rate in Sweden, what events, regulations, developments in international / national level may have contributed to the development of employment (fill in the timeline)? It can be about events or processes regarding policies, but also other areas that you think are relevant (post-it discussion)
- What do you think of when you see the diagram of the employment rate in the various groups (notable parts of the graph) against the background of developments that you mentioned?
- Is it in line with your experience and knowledge?
- In what ways may the development of employment depend on issues you mentioned in the timeline (ideas for explanations, associations)?

<u>1b) presented results (10 min): Employment Charts for SE, DK, NL, UK, compared</u>

- What do you think when you see this European picture where all graphs show a negative trend for the target group?
- What do you think about Sweden's position in the European comparison?

00: 50-01: 10: Discussion part II: poverty risk

<u>Presented results (20 min): Poverty Risk SE, DK, NL, UK</u>

- What do you think of when you see the diagram of the risk of poverty in the different countries (notable parts of the diagram)?
- Is it in line with your experience and knowledge?

- Do you see a connection between poverty and issues you mentioned that you mentioned on the timeline? What then do you think causes the evolution of poverty risk?

01: 10-01: 30 Discussion part III: policy implications

Presented results (20 min): empty logic model

- What regulations and actions are hiding behind the concepts ALMP, flexibility, economic security? (still possible to add something)
- We would like to ask you to fill in the consequences you think that policies affecting economic security, flexibility and active labor market policies may have on employers and employees.
- We have come to this model (show), what do you think? Does it help with carrots / sticks?

01: 30-01: 45: Discussion part IV: flexicurity in Sweden

Presented results (15 min): 4-field diagram with countries DK, NL, UK, without SE

- Position Sweden in the table according to how flexible and secure you feel the Swedish labor market is, and state why you place Sweden where you see it in the table.
- Has there been development over the last 20 years that you can describe and what do you think the future development will look like?

What do these findings say on how to reduce inequalities in health in society? Have we selected the right target group (low edu LLI) and indicators data in that regard?

01: 45-02: 00: End of the Workshop: round up

- What do you take home of what we've talked about today (the most important)? Please summarize briefly.

• What should we take home as a form of research assignments, i.e. questions, focus, and the like for future research from your perspective as representatives of [name of public or stakeholder group]?
