# Supplementary material for: Involving the public in epidemiological public health research: a qualitative study of public and stakeholder involvement in evaluation of a population-wide natural policy experiment
Source: BMJ Open. 2018 Apr 20;8(4):e019805. doi: 10.1136/bmjopen-2017-019805 (PMC5914713; doi:10.1136/bmjopen-2017-019805)
Supplement: Author's manuscript [file bmjopen-2017-019805.draft_revisions.pdf]

Impact of public involvement in interpreting research findings: the effect of labour market policies on inequalities in employment and health outcomes

Journal:	*BMJ Open*
Manuscript ID	bmjopen-2017-019805
Article Type:	Research
Date Submitted by the Author:	09-Oct-2017

Complete List of Authors:	Anderson de Cuevas, Rachel; University of Liverpool, Public Health & Policy<br>Nylén, Lotta; Karolinska Institutet, Department of Public Health Sciences, Division of Social Medicine<br>Burström, Bo; Karolinska Institutet, Department of Public Health Sciences, Division of Social Medicine<br>Whitehead, Margaret; University of Liverpool, Public Health

**Primary Subject Heading**:	Public health
Secondary Subject Heading:	Research methods
Keywords:	QUALITATIVE RESEARCH, PUBLIC HEALTH, SOCIAL MEDICINE

SCHOLARONE™
Manuscripts

For peer review only

For peer review only - http://bmjopen.bmj.com/site/about/guidelines.xhtml

Impact of public involvement in interpreting research findings: the effect of labour market policies on inequalities in employment and health outcomes

Authors:

Rachel Anderson de Cuevas[1†], Lotta Nylén[2], Bo Burström[2*], Margaret Whitehead[1]

Affiliated institutions:

[1]Department of Public Health, University of Liverpool, Liverpool, Merseyside, United Kingdom; [2]Department of Public Health Sciences, Karolinska Institute, Stockholm, Stockholm County, Sweden.

†Corresponding Author Pre Publication, Post Doctoral Research Associate, Department of Public Health and Policy, Institute of Psychology, Health and Society, 3rd floor, Whelan Building, Brownlow Hill, Liverpool, United Kingdom L69 3GB, Email: rma@liverpool.ac.uk

*Corresponding Author Post Publication, Professor of Social Medicine, Division of Social Medicine, Dept. of Public Health Sciences, Karolinska Institute, Department of Public Health Sciences (PHS), K9, Social Medicin, 171 77 Stockholm, Sweden, Email: Bo.Burstrom@ki.se

Keywords: health inequalities; social determinants of health; Europe; public involvement; flexicurity.

Word count: 3,995.

For peer review only

Abstract

Objectives: This project aimed to: represent the concerns of groups in weak positions in the labour market, in an evaluation of the effect of Flexicurity policies on the social differentials in employment and poverty risk of people with limiting longstanding illness; and assess the impact of public involvement on the interpretation of research findings. Public involvement in research is considered good practice by European funders; however evidence of its research impact is sparse, particularly in relation to health inequalities.

Setting: Stockholm County, Sweden.

Participants: Key contacts were identified from the research group's existing network to provide access to stakeholders using a snowballing technique. Four public groups (17 participants) were recruited: 1) three trade unions; 2) the Swedish national employment agency; 3) an employers' association and 4) politicians from the ruling coalition and opposition parties, who sat on a national labour market committee.

Outcome measures: The impact of involving the public on the interpretation of study findings was assessed in three principal ways: 1) based on a record of the public's contribution; 2) comparison with the researchers' interpretation and 3) the researchers' perspectives of the public involvement process, together with the comments of academic peers.

Results: The public provided important insights and context to the events and policies influencing the declining employment of people with activity limitations. The perspective of the public representatives differed between groups and to that of the researchers. Unionists highlighted the erosion of universal social security systems and deregulation, while the employers' association and some politicians emphasised global competition. The

For peer review only

employment agency mentioned changes to the size and composition of the group and alteration in workplace attitude.

Conclusions: Participants offered valuable insights into the complexities of the labour market and their effects on health inequalities and helped re-shape researchers' interpretation of findings in subtle ways.

Strengths and limitations of this study

- The study is one of the few reports of public involvement in health inequalities research and furthermore in a European country outside the UK, where the concept is not widely recognised.
- This is also the first report of involving the public in the interpretation of quantitative data, to the researchers' knowledge.
- The research group did not engage individuals with activity limitations due to health problems about their experience of labour market policies or barriers to accessing the labour market.
- The public were involved in one discrete phase of the research process, rather than throughout.

For peer review only

Introduction and background

Involving the public in health and social research is regarded by funding bodies and policy makers as an integral part of good practice and democratization of the research process (1). There is growing recognition that lay people have valuable expertise that should inform the research process, as well as a right to be more actively involved in the design and outputs of research that is about and for them. Increasingly, research funding bodies also require researchers to demonstrate the value of public involvement by evidencing its impact (2). We sought to involve the public in research into the health inequalities bound up with labour market policies and to assess what impact that involvement made to the outcomes of the research. As part of the Demetriq project, we conducted a study of the impact of 'flexicurity' (labour market flexibility and the provision of social security) on the employment chances of people with limiting longstanding illness (LLI) according to their socioeconomic status (3). As such, we sought to involve trade unions and other public bodies working closely with return-to-work policies in a discrete phase of the research project: the discussion and interpretation of research findings.

'Flexicurity' and the employment of people with a disability or illness

There is a general rise across Europe in the numbers of disabled and chronically ill people of working age that are not in employment. This is a matter of growing concern for governments due to its adverse consequences for public health and potential to exacerbate health inequalities, as well as its effect on increasing social security expenditure (4). Ageing populations and a raised retirement age in several countries mean that the proportion of the working age population leaving the labour force due to ill-health is expected to rise further (4, 5).

For peer review only

In Sweden, the proportion of the population receiving sickness or activity compensation has trebled in the last 30 years (6). The combined cost of sick leave and disability pensions equates to approximately 4% of Swedish GDP and nearly 8% of the working age population receive disability pensions (4). The situation in Denmark and Norway is similar (8 and 10%, respectively), while in the UK, more than 2.6 million people receive incapacity-related benefits, accounting for a quarter of the social security budget and 1.5% of GDP (7).

Being outside the labour market is likely to add to the social and economic exclusion of people with a disability or illness, as income, opportunities to participate fully in society and quality of life decrease (4, 5). People in poor health have lower employment prospects than the general population and these prospects decrease in line with declining socioeconomic position (8, 9). The social gradient in the employment of people with disabilities may serve to widen health inequalities, since the more disadvantaged who already have a higher prevalence of ill health will be further disadvantaged by the negative effects of unemployment (5).

European countries have undertaken a range of initiatives which may affect the ability of chronically ill and disabled people to participate in the labour market (4). Population-wide policies centre on the flexibility and de-regulation of the labour market and disability-related benefits. These policies have generated considerable debate about their possible impact on the employment opportunities of chronically ill and disabled people.

Flexibility of employment conditions is considered to have contributed to higher levels of job insecurity and to have disproportionately affected those who find themselves in the most vulnerable positions in the labour force (including women, the low skilled and people with disabilities) (10). Since labour market deregulation is considered an essential strategy

For peer review only

to redress the economic downturn, 'flexicurity' has been proposed as an alternative; compensating for increased flexibility with provisions of security (*ibid*). Flexicurity policies aim to integrate labour market flexibility with social security and active labour market policies, within a climate of competition and rapid technological change (*ibid*). (Denmark and the Netherlands are often cited as exemplars of flexicurity in its most developed form). Yet, little is known about the consequences of flexicurity for health and the social determinants of health.

Study design and methods

The study aimed to involve relevant public bodies in explaining, challenging and interpreting findings from a research project evaluating the effect of 'flexicure' labour market policies on the social differentials in employment of people with a limiting longstanding illness and to assess the difference public involvement made to the researchers' interpretation and discussion of findings. The Demetriq project investigated the impact of labour market policies, which individual European countries had adopted over time, on the (re-) employment chances of people with activity limitations due to health problems, and examined the relationship between socioeconomic status and employment chances. Researchers undertook policy analysis and interrogated country-specific data on employment rates in different groups, using a range of statistical analytic methods suited to Natural Policy Experiments, published separately (see for example (11)).

The study component reported here comprised focused workshops with key stakeholder groups to elicit fresh interpretations of selected research findings, using the process developed by Petticrew and Whitehead (12, 13). The planning, process and outcomes of involving the public was recorded and evidenced in parallel to the activity. Preliminary

For peer review only

research results were presented to public representatives in graphical form during focused workshops, to elicit stakeholders' perspectives on selected research findings. Structured interviews were conducted with researchers before and after the public involvement activity to explore researchers' developing attitude towards public involvement and their perceptions of its impact on the research. The process of selecting key research findings and the research group's evolving approach to public involvement was observed during regular team meetings. Lastly, a focus group was held with an interdisciplinary research interest group on Equity and Health Policy Analysis at the host institution, to allow critical peer discussion of the public's contributions and involvement process.

The impact of involving the public on the interpretation of study findings was assessed in three principal ways: based on a record of the public's contribution; comparison with the researchers' interpretation and the researchers' perspectives of the public involvement process, together with the comments of academic peers. The researchers' interpretation of study data was recorded in technical reports and presentations prior to the public involvement activity.

Two researchers with expertise in qualitative research methods and public engagement, who were independent from the primary study, designed and led the public involvement activity and conducted the analysis. An independent moderator facilitated the workshops.

For peer review only

Identifying and engaging the public

The project engaged members of the public who played a key role in developing, implementing or interpreting return-to-work policies, and especially people who represented and understood the concerns of people in vulnerable positions on the labour market. Key contacts were identified from the research group's existing network to provide access to stakeholders using a snowballing technique. Members of the public were sent invitation letters, an outline of the research project and the objectives of the public involvement activity. Interested stakeholders were given further information about the research and engagement activity through telephone conversations, face-to-face meetings and written correspondence.

Four public groups were recruited: 1) three trade unions representing blue- and white-collar workers and academics; 2) the Swedish national employment agency; 3) an employers' association for private sector business and industry and 4) politicians from the Moderate, Centre (the ruling coalition), Social Democratic, Left and the Green Party (in opposition), who sat on a national labour market committee.

Description of public involvement activity

Selected findings were presented at 2 hour focused workshops after Petticrew and Whitehead (12, 13), held separately with each public group at their workplace during June and August 2014. Two to six participants attended each session, with a total of 17 participants. Findings were presented in diagrammatic form (as described in Box 1). One researcher moderated the discussion and 1 or 2 members of the core research team presented the findings. A standardised topic guide was followed (Appendix 1), with additional questions tailored to suit each group's expertise and interest. Focused

For peer review only

workshops were conducted and transcribed in Swedish and selected quotations translated into English.

Box 1: Description of material presented to the public

The public participants were presented with a graph showing trends in Swedish employment rates of people with a limiting longstanding illness (LLI) and healthy people stratified by education from 1990 to 2011, constructed from the Swedish Survey on Living Conditions and a blank timeline for the same period. The public participants were asked to chart important events or trends during this period on the timeline and to interpret developments in the employment of people with LLI and healthy people in the light of these events. Participants then compared graphs presenting differences in the employment rate of those with and without LLI and with high and low educational levels in Sweden, Denmark, the Netherlands and the United Kingdom for the period 2005 – 2011 from EU SILC data (European Union Statistics of Income and Living Conditions, EUROSTAT) and commented and reflected on the situation in Sweden compared with the other 3 countries. The differential rate of 'poverty risk' (<60 per cent of median income) among non-employed/employed people with LLI and employed healthy people was also presented in graphical form (EU SILC) and the public was asked to interpret changes in poverty risk in light of the key socio-political or economic events they had charted on the timeline. Lastly, participants considered the categorisation of the UK, Netherlands and Denmark in relation to their degree of employment protection and economic security (Flexicurity) in a 2x2 matrix and reflected where to best position Sweden and the trajectory of its position over time.

For peer review only

Analytic framework

Researchers compared the public's interpretation of trends affecting the employment of people with chronic health conditions with that of the research group. Full transcripts of the digital recordings of the focused workshops were coded and categorised, employing the principles of content analysis, for the data set as a whole and separately for each workshop (14, 15). Thematic analysis of the moderator's notes and the transcripts drew out key themes from each workshop to form a summary of themes across the workshops and to compare the insights of the 4 different public groups between workshops (16). Transcription and analysis were undertaken alongside the public involvement activity as part of an iterative process which informed each phase of data collection (17). The record of process, which comprised observation of research meetings, interviews with researchers and critical peer reflection, provided important context for assessing the contribution of the public (18).

Research ethics

Ethical clearance was not required under Swedish law on ethical review (refer to Lag 2003:460), as the information collected was not considered to constitute sensitive personal information (§3 in the Law). Stakeholders participated as collective members of their employing institution, not as individuals or the subjects of the research. All gave informed consent to being interviewed and for the interviews to be recorded and transcribed.

For peer review only

Results

Participating public bodies comprised professional women and men age 40 to 60 years (17 participants). All groups commented on the decline in the employment rate of low educated people with LLI over time and offered different explanations for this development (Table 1).

For peer review only

Table 1. **Summary of results from workshops.**

What explains lower employment rate in target group?	Which policy changes have occurred?	What explains increasing rates of relative poverty among non-employed?	Has Sweden moved to flexicurity
Globalisation: higher demands on workers, higher education requirements. Economic recessions early 1990s, early 2000 and 2008. (agreement)	Fighting inflation instead of unemployment, to be competitive (TU). De-regulation of many sectors (e.g. transport, construction) (TU)	Increasing market incomes, tax reductions. (P) Stagnant levels of unemployment and social assistance benefits. (P)	Varies between individuals – if you are part of unemployment insurance and collective agreements, you are secure; otherwise not. Labour market not flexible (E).
Changing labour market: New complex technology – increased demand on workers. Reduced demand for unqualified workers (agreement)	Less emphasis on vocational training and life-long learning (TU, EA)	Security has moved to other arenas – collective agreements and individual choices more important (TU, E)	Flexibility for employers has increased. Security has become differentiated – not general. Depends on where you are in the system (TU)
Changing composition of group with low education and LLI – more low educated immigrants with refugee background. (E, EA)	Social security reform in 2008 - reduced duration of sickness absence, restrictions on disability pension (agreement).	Fewer eligible for disability pension, fewer covered by unemployment insurance (EA)	Decreased economic security, not increased flexibility for the employer. Varies between different employers and white/blue collar trade unions, depends on which sector. (EA)
Increasing levels of mental ill health – more difficult to find jobs, lock-in effects. Focus on individual's efforts. (EA)	Reduced economic security to incentivize work (EA)	Reduced economic security to incentivize work (EA)	Politicians divided on the issue – most agree that flexibility is high; economic security is also considered high, but social democrats and green party think security has been lowered (P).
Social insurance reform shifted large groups from social insurance to unemployment	Security has moved to other arenas – collective agreements and individual choices more important (TU, E)	Increased unemployment insurance fee – lower participation (TU,P)	
Target group less likely to be employed (E)	Extended period for employers to pay sickness benefits (E).	More unemployed rely on social assistance (EA, TU)	

E = employers; P = politicians; TU = trade unions; EA = employment agency.

Explanations for the lower employment rate of low educated people with LLI in Sweden

Public participants put forward the economic recessions of the early 1990s, early 2000s and 2008, as explanations for the low employment rate of low educated people with LLI in Sweden: *"That's how it is. We know that in every recession […] they fall further and further down. They are last in line."* (Trade unionist). Many considered economic cycles to have had a greater influence on employment rates than national policies. The employer's association and some of the politicians emphasized that the pressures of global competition had led to outsourcing of low-skilled production tasks abroad, placed greater exigencies on workers in employment and removed demand for low-skilled workers with health problems. As one politician put it: *"… we have a labour market which does not have room for these people; as [was the case] … in the 1990s …"*

The 2008 reform of Swedish social insurance limited the time allowed for sickness absence and shifted a large group of people from social insurance to unemployment benefits. This was noted by the employment agency: *"those expelled from the insurance… the first batch … - [I] think it was 4th January 2010 - … 14,000 people suddenly came to the employment agency."*

The composition and size of the low educated group with LLI was also seen to have changed. The group had become smaller, as a result of the rise in the educational level of the general population and included a higher proportion of immigrants and people with mental ill-health. This was seen as another explanation for the group's lower employment rate: *"A large proportion of recent immigrants are very low educated or illiterate and of course they have greater difficulties [in securing employment] …"* (Employment agency).

For peer review only - http://bmjopen.bmj.com/site/about/guidelines.xhtml

Another employment agency employee said that it was harder for people with functional disabilities resulting from mental illness to find jobs: *"… it is easier to adjust physically … but if you have ADHD and forget your appointment time …"*

A change in work place attitude towards people with disabilities was also mentioned as a factor contributing to the difficulty for low educated people with LLI to find employment: *"The climate has sort of become tougher"* (*Employment agency*).

What are the key policy changes in Sweden that have influenced the employment of this group from 1990-2010?

Trade unionists suggested that changes in economic policy underpinned some of the trends in the employment rate of low educated people with activity limitations due to health: *"I believe that the higher structural unemployment that results from prioritizing low inflation over low unemployment enables employers to pick and choose. And then they pick the high educated … rather than the low educated."* Deregulation of many different sectors was also mentioned:

"[The] taxi [industry] in Sweden is the most de-regulated in the world."

Trade unionists felt that that there had previously been much more emphasis on providing vocational training and continuing education for people with LLI who were not employed, whereas today individuals – particularly blue collar workers – are simply urged to look for and obtain a job: *"… you would think our government could step in and make sure these groups get at least an equal chance and support blue collar workers."*

Some interviewees, including trade union representatives, described how universal social security systems had been eroded over time. Unemployment insurance contributions had

For peer review only - http://bmjopen.bmj.com/site/about/guidelines.xhtml

increased substantially, resulting in many employees leaving the scheme. Unemployment benefit levels had stagnated or been lowered and social assistance benefits had plateaued. In parallel with the erosion of universal social security systems, collective agreements between employers and employees had changed, partly to compensate for this phenomenon. As one trade unionist put it: *"Security has moved to other arenas"*. The revised collective agreements were seen to have a significant impact on the employment and working conditions, social protection and retraining opportunities of the employees whom they covered. The terms and conditions of the collective agreements however varied for white and blue-collar workers; blue collar workers having a more restricted choice of training and fewer long-term training opportunities.

Employers mentioned another policy influence on people with LLI; an extension to the period of time an employer is responsible for paying sickness benefits for an absent employee, thereby increasing the cost to the employer. This policy change may have been detrimental to the employment chances of persons with LLI. Other reforms to social security regulations, including the aforementioned restriction to the duration of sickness absence and also to eligibility for disability pension, were considered by many interviewees to be important policy changes which incentivized work: *"It's tougher to be an outsider [to the labour market] today than [it was] 6 years ago … This is partly due to a political desire to strengthen the incentive to work"* (Employment agency).

What explains increasing rates of relative poverty among non-employed persons with limiting longstanding illness in different European countries?

Workshop participants were shown results comparing the prevalence of relative poverty among employed and non-employed people with limiting longstanding illness in Sweden, Denmark, the Netherlands and the United Kingdom, for the years 2005 and 2010,

For peer review only - http://bmjopen.bmj.com/site/about/guidelines.xhtml

respectively. In Sweden, the poverty rate had nearly doubled from 2005 to 2010 albeit from a very low starting point, whereas increases in the other countries, which started with higher poverty rates in 2005, were smaller. Some politicians questioned the validity of the measure "relative poverty" (defined as having an income below 60 percent of the median national income): *"… it describes an increasing income difference in society, but it says nothing about whether poverty has increased. These are two completely different things."* Other interviewees attributed the increasing rates of relative poverty directly to policies: *"… it has been a deliberate policy … to increase the differences in income according to whether you work or do not work, … so you notice the change if you start working"* (Employment agency).

The social insurance reform of 2008 had excluded many non-employed people with LLI from sickness benefit, yet many were not on the unemployment register. The issue was how they made their living: *"… they are somehow supported by relatives … The group with mental problems … seek social assistance more than others"* (Employment agency).

The increase in unemployment insurance contributions (due to a change in government policy in 2007) led to many employees leaving the insurance and was also put forward as an explanation for rising rates of relative poverty among this population group: *"… there are many unemployed people who are not part of the unemployment insurance scheme at all, and who don't get anything …"* (Employment agency).

Has Sweden moved to flexicurity?

Workshop participants were asked whether they considered that the policy changes that had taken place from 1990 to 2010 had moved Sweden to qualify as a 'flexicure' country and were asked to place Sweden in a matrix of high/low flexibility and high/low economic

For peer review only - http://bmjopen.bmj.com/site/about/guidelines.xhtml

security (Figure 1). (Denmark, the Netherlands and the UK were positioned by the researchers). Opinions were divided. One trade unionist commented: *"The whole of [Swedish] working life has slipped towards the UK [position, with high flexibility and low economic security] …"* Another trade unionist also thought that flexibility had increased: *"In Sweden, we have perhaps the highest flexibility out of many countries, to fire people … It is incredibly liberal … We belong to high flexibility."*. Employers, on the other hand, considered that flexibility was low, but that the degree of economic security differed according to whether or not an employee was part of unemployment insurance and covered by collective agreements. For those who did not belong to these schemes, security was lower than before: *"… the universal welfare system has been hollowed out since the 1990s"* (Employer). Many trade unionists and employers referred to a two-tier system, in which the situation for 'insiders' to the labour market - those who have had a job for some time, who are part of the unemployment insurance scheme and are covered by collective agreements - was much better than for those outside these arrangements. Most politicians commented that Sweden had high flexibility and high security, having moved from lower flexibility and even higher economic security. One politician spoke of: *"… a divided labour market … There are those who have security and those who do not …"*

For peer review only - http://bmjopen.bmj.com/site/about/guidelines.xhtml

Fig 1: Matrix showing the different categorizations of Sweden's degree of Flexicurity by the 4 public groups

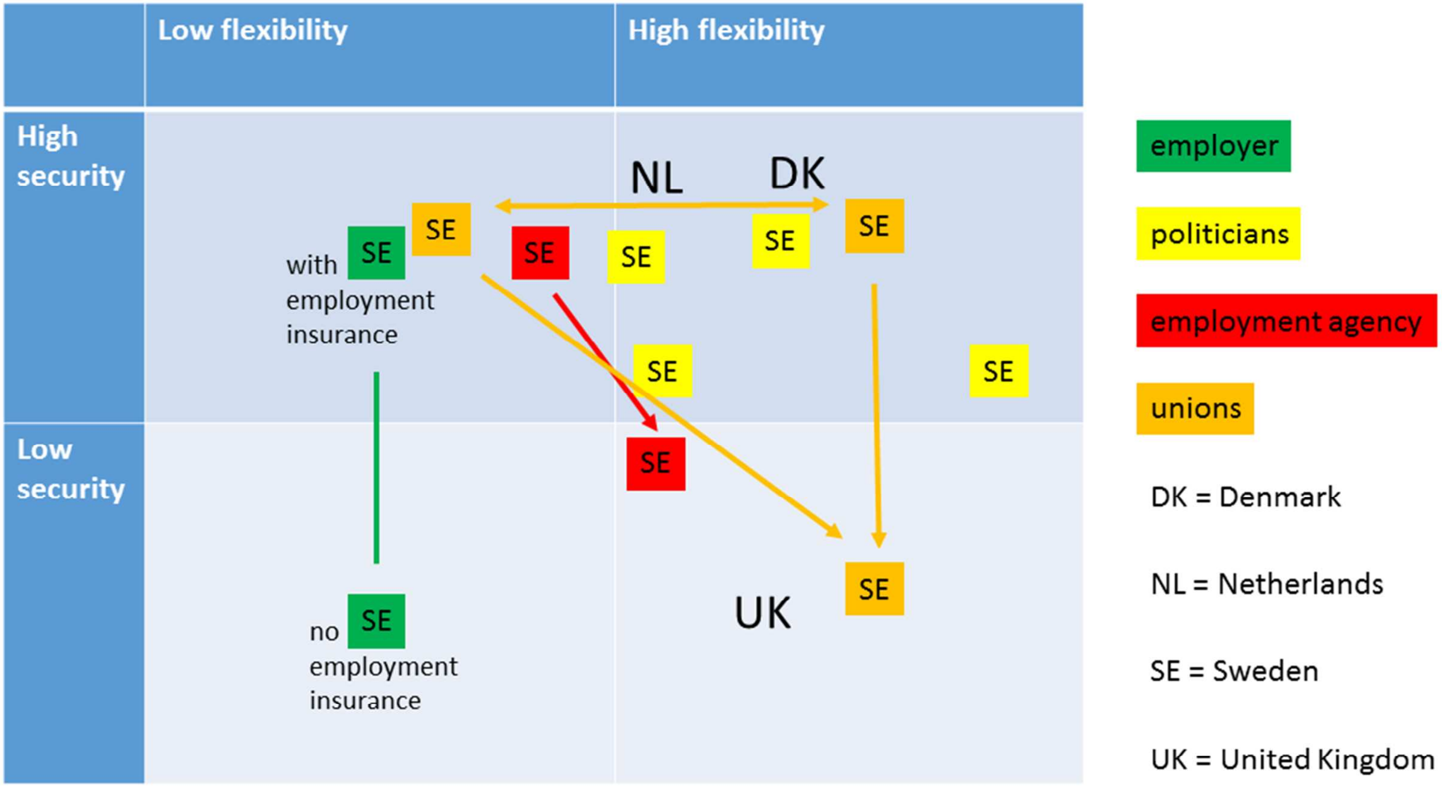

For peer review only - http://bmjopen.bmj.com/site/about/guidelines.xhtml

For peer review only

For peer review only - http://bmjopen.bmj.com/site/about/guidelines.xhtml

Influence of public involvement on the researcher's interpretation of findings

The public's interpretation of the research findings influenced the researchers' thinking in several ways. Firstly, the public provided fresh insights into the events and policies influencing the declining employment of low educated people with LLI that had not been considered by the researchers. Secondly, stakeholders provided important context to the experience of people in vulnerable positions in the labour market. This added layers of complexity to the researchers' interpretation of events, leading to the construction of multiple causal explanations for the increasing rates of relative poverty and decline in employment of the target group and to a more nuanced analysis. Thirdly, the participants' perspective differed between and also within the public groups, which highlighted to the research group that a single phenomenon, such as the degree to which Sweden is considered a Flexicure country, is also a matter of interpretation, as well as a measurable concept.

In terms of policy insights, for example, trade unionists emphasised the importance of collective agreements in providing economic security for represented employees, leaving employees who were not part of collective agreements worse off and drew attention to the fact that collective agreements were more favourable for white-collar workers.

The employers' contribution reinforced to the researchers that global economic and labour forces are also important influences on employment. Employers presented the reduced employment rate among low educated people with LLI to be a consequence of the effects of globalisation, namely increasing competition, changing labour markets and demands for a more highly skilled workforce. Employers reasoned that there are few jobs available for

For peer review only - http://bmjopen.bmj.com/site/about/guidelines.xhtml

people with limited work capacity, social and communication skills, as low skilled jobs have moved from the manual to the service sector. The researchers' analysis had found that high overall employment in a country was a predictor of higher rates of return-to-work among people with LLI (19). The researchers therefore concluded that low educated people with LLI obtain employment when the economy is healthy and overall employment is high, since they are the "*last to be hired and first to be fired*", as expressed by a trade unionist. The researchers were surprised at the politicians' suggestion that national policies could not mitigate the effect of economic cycles: "*I was surprised to see that they didn't, as policymakers, think that policies made a difference! That's a kind of pragmatic view on [the] part of policymakers. I thought they would think that policies made more of a difference*".

The public – especially the unions – highlighted the complexity of national and local policies, macroeconomic forces and their effects. Participants emphasised that populations have very different experiences of the labour market, depending on their socioeconomic, health and occupational status, and whether or not they are established in the labour market or subscribed to the unions' unemployment insurance schemes.

Each public group positioned Sweden differently in relation to its degree of flexibility and security. The difficulty the public had in characterising Sweden in terms of Flexicurity and the difference of opinion between the stakeholder groups in interpreting Flexicurity, made the researchers realise that the policy is conceived differently within and between countries and the experience of different actors also varies. The differences in perception between the groups was of considerable interest to researchers, who felt this warranted further analysis and might provide material for further investigation. At the same time, these

For peer review only - http://bmjopen.bmj.com/site/about/guidelines.xhtml

differences were seen to pose challenges for impact assessment, since researchers may not be comparing like with like.

Research context and attitudes towards public involvement

This was the first time that the research group had undertaken public involvement activities, whose purpose was conceived to be to validate, check and enhance their preliminary interpretation of findings. The public's reaction and comments confirmed to the researchers that the public shared the researchers' broad understanding of the labour market context and the economic and political events shaping it. The public's perspective, however, differed from that of the researchers regarding the characterisation of Sweden's labour market and social security policies and this made the researchers reflect on the uncertain status of research evidence in informing policymaking processes. The views of the researchers and the public also partly diverged with respect to the measures that should be taken to address unemployment, financial insecurity, low education and the exclusion of people with LLI.

Impact of public involvement on the research process

The several different characterisations of Swedish policies relating to flexibility and security and their changing trajectory over time, provided researchers with additional material for reflection and analysis, which led to further avenues for investigation. The changing composition of the group 'low educated people with LLI', which the public raised, was also considered to be an interesting question for future research. The researchers were enthused by the public's general level of engagement in the topic, which led to the identification of potential collaborations. The employment agency, for example, suggested their data could be utilised to investigate the employment and economic situation of

For peer review only - http://bmjopen.bmj.com/site/about/guidelines.xhtml

subgroups, such as people with LLI and psychological illnesses. Despite its acknowledged value, the public involvement activity also represented a substantial amount of work for the research group, which had not been built in to research planning.

Discussion

This is one of the few reports of public involvement in health inequalities research and furthermore in a European country outside the UK, where the concept is not widely recognised. To our knowledge, this is also the first report of involving the public in the interpretation of quantitative data (20).

The research group largely considered the public involvement activity to have been a success, despite being time intensive. The activity could be classified as having comprised fairly low level participation, as the public were participants in a single phase of the research (21). The researchers very much valued the public's contributions, as they highlighted what were the priority issues for different public groups, raised issues for future research and demonstrated willingness for future collaboration.

Reservations have been expressed elsewhere about the difficulties of involving the public in research that is highly technical (22) and some might include the evaluation of Natural Policy Experiments for their impact on health inequalities in this bracket. The material presented to the public in this project represented a small selection of the overall study findings and was presented in graphical form. The public was not presented with raw data, nor informed about data availability, decisions relating to study design, selection of indicators or methods of statistical analysis. The researchers presented the public with "*big trends over time*", on the basis that this would be material that they could easily react to in

For peer review only - http://bmjopen.bmj.com/site/about/guidelines.xhtml

the short time available. Members of the public demonstrated a high level of expertise and a capacity to engage with the intricacies of policy developments, changes to the economy, the country's demographic profile and global market forces, from both a practical and theoretical perspective.

The stated benefits of involving the public in research have often been assumed rather than evidenced (23, 24), since reporting of public involvement in research to date has tended to concentrate on *process* (2). This study has therefore attempted to report public involvement activity comprehensively and to assess its impact in order to add to the evidence base (25). The study was designed to obtain a clear distinction between the researchers' perspective of the study findings before and after public involvement, as the researchers were aware that isolating its unique effects is challenging (22).

Study limitations

The research group did not engage individuals with activity limitations due to health problems. Involvement of people with direct experience of labour market policies or barriers to accessing the labour market, would strengthen a future research project in this area (21).

The public, in this case, were involved in one discrete phase of the research process. There is some evidence to suggest that involving the public throughout the research project (as well as long-term involvement and continuity of membership of the public beyond a single research project) enhances impact (Staley 2009, Staniszewska 2009, PiiAF 2013). The research group felt that involving the public from the research concept and design stage in future research, would facilitate engagement of the target group and maximise their

For peer review only - http://bmjopen.bmj.com/site/about/guidelines.xhtml

potential contribution. Most participant groups expressed an interest in future collaboration, which might also facilitate engagement with people with LLI.

Conclusion

The insights and reflections the public offered added depth and subtlety to the researchers' interpretation of the research findings and highlighted the issues perceived to be of most public relevance. In addition, the public raised questions that could not be answered by the current study, opening the door to future research.

For peer review only

For peer review only - http://bmjopen.bmj.com/site/about/guidelines.xhtml

Contributorship statement:

MMW and BB conceived the research study. LN and RMAdC designed and led primary data collection and analysed the data. RMAdC and BB wrote the first draft and together with MMW and LN interpreted the data. All authors contributed to the final draft.

Competing interests:

The authors have no conflicts of interest to declare.

Funding:

This research was funded by an European Union FP7 grant DEMETRIQ (developing methodologies to reduce inequalities in the determinants of health) (Grant Reference: 278511), http://www.demetriq.eu/. The funders had no role in study design, data collection and analysis, decision to publish, or preparation of the manuscript. The authors of this paper alone are responsible for the views expressed in this publication which do not necessarily represent the decisions or policies of the EU or their institutions.

Data sharing statement:

Transcripts of the focussed discussions, field notes and observations and in-depth interviews with research group members are in Swedish and available on request from the corresponding author.

For peer review only - http://bmjopen.bmj.com/site/about/guidelines.xhtml

References

1. Boote J, Baird W, Beecroft C. Public involvement at the design stage of primary health research: a narrative review of case examples. Health policy (Amsterdam, Netherlands). 2010;95(1):10-23. Epub 2009/12/08.
2. Snape D, Kirkham J, Britten N, Froggatt K, Gradinger F, Lobban F, et al. Exploring perceived barriers, drivers, impacts and the need for evaluation of public involvement in health and social care research: a modified Delphi study. BMJ open. 2014;4(6):e004943. Epub 2014/06/19.
3. Mackenbach J, Whitehead M, Burström B, Stuckler D, Judge K, Platt S, et al. DEMETRIQ (developing methodologies to reduce inequalities in the determinants of health). European Union 2012.
4. Whitehead M. Disability and employment: lessons from natural policy experiments. European journal of public health. 2010;20(4):371-3. Epub 2010/07/28.
5. Clayton S, Barr B, Nylen L, Burstrom B, Thielen K, Diderichsen F, et al. Effectiveness of return-to-work interventions for disabled people: a systematic review of government initiatives focused on changing the behaviour of employers. European journal of public health. 2012;22(3):434-9. Epub 2011/08/06.
6. Effective return to work. Reforms. Stockholm: Government Offices of Sweden, 2008.
7. Clayton S, Bambra C, Gosling R, Povall S, Misso K, Whitehead M. Assembling the evidence jigsaw: insights from a systematic review of UK studies of individual-focused return to work initiatives for disabled and long-term ill people. BMC public health. 2011;11:170. Epub 2011/03/23.
8. Burstrom B, Holland P, Diderichsen F, Whitehead M. Winners and losers in flexible labor markets: the fate of women with chronic illness in contrasting policy environments--Sweden and Britain. International journal of health services : planning, administration, evaluation. 2003;33(2):199-217. Epub 2003/06/13.
9. Burstrom B, Whitehead M, Lindholm C, Diderichsen F. Inequality in the social consequences of illness: how well do people with long-term illness fare in the British and Swedish labor markets? International journal of health services : planning, administration, evaluation. 2000;30(3):435-51. Epub 2000/12/08.
10. Afzal Z, Muntaner C, Chung H, Mahmood Q, Ng E, Schrecker T. Complementarities or contradictions? Scoping the health dimensions of "flexicurity" labor market policies. Int J Health Serv. 2013;43(3):473-82. Epub 2013/09/27.
11. McAllister A, Nylen L, Backhans M, Boye K, Thielen K, Whitehead M, et al. Do 'flexicurity' Policies Work for People With Low Education and Health Problems? A Comparison of Labour Market Policies and Employment Rates in Denmark, The Netherlands, Sweden, and the United Kingdom 1990-2010. International journal of health services: planning, administration, evaluation. 2015;45(4):679-705.
12. Petticrew M, Whitehead M, Macintyre SJ, Graham H, Egan M. Evidence for public health policy on inequalities: 1: the reality according to policymakers. Journal of epidemiology and community health. 2004;58(10):811-6. Epub 2004/09/15.
13. Whitehead M, Petticrew M, Graham H, Macintyre SJ, Bambra C, Egan M. Evidence for public health policy on inequalities: 2: assembling the evidence jigsaw. Journal of epidemiology and community health. 2004;58(10):817-21. Epub 2004/09/15.
14. Robson C. Real World Research. 2nd edition ed. Oxford: Blackwell; 2002.

For peer review only - http://bmjopen.bmj.com/site/about/guidelines.xhtml

15. Graneheim UH, Lundman B. Qualitative content analysis in nursing research: concept, procedures and measures to achieve trustworthiness. Nurse Education Today. 2004;24(105-112).
16. Braun V, Clarke V. Using thematic analysis in psychology. Qualitative research in psychology. 2006;3(2):77-101.
17. Ritchie J, Lewis J, editors. Qualitative research practice: a guide for social science students and researchers. London: Sage Publications Limited; 2003.
18. Gradinger F, Britten N. Values associate with public involvement in health and social care research: a narrative review. Health Expectations. 2013.
19. Backhans M, Mosedale S, Whitehead M, Burstrom B. What is the impact of flexicurity on the chances of entry into employment for people with low education and activity limitations due to health problems? A comparison of EU countries using Qualitative Comparative Analysis (QCA). In manuscript.
20. Staley K. Exploring impact: Public involvment in NHS, public health and social care research. Eastleigh: INVOLVE, 2009.
21. Beresford P. User involvement, research and health inequalities: developing new directions. Health Soc Care Community. 2007;15(4):306-12. Epub 2007/06/21.
22. Barber R, Boote JD, Parry GD, Cooper CL, Yeeles P, Cook S. Can the impact of public involvement on research be evaluated? A mixed methods study. Health expectations : an international journal of public participation in health care and health policy. 2012;15(3):229-41. Epub 2011/02/18.
23. Nilsen ES, Myrhaug HT, Johansen M, Oliver S, Oxman AD. Methods of consumer involvement in developing healthcare policy and research, clinical practice guidelines and patient information material. The Cochrane database of systematic reviews. 2006(3):Cd004563. Epub 2006/07/21.
24. Staniszewska S, Adebajo A, Barber R, Beresford P, Brady LM, Brett J, et al. Developing the evidence base of patient and public involvement in health and social care research: The case for measuring impact. International Journal of Consumer Studies. 2011;35(6):628-32.
25. Brett J, Staniszewska S, Mockford C, Herron-Marx S, Hughes J, Tysall C, et al. Mapping the impact of patient and public involvement on health and social care research: a systematic review. Health expectations : an international journal of public participation in health care and health policy. 2014;17(5):637-50. Epub 2012/07/20.

For peer review only

For peer review only - http://bmjopen.bmj.com/site/about/guidelines.xhtml

S1 Appendix 1: Focused Workshop standard topic guide

Public

WS-leader Expert (introduce Demetriq + results), Moderator (questions, discussion)

Dual audio recording (mp3 + smartphone)

Demetriq = Developing methodologies to reduce inequalities in the determinants of health

Schedule (times are not exact, changed flexibly according to the workshop's progress)

00: 00-00: 15/20 Introduction

Introductory Workshop - Moderator; hello-hello, my name is ... and do ... (about 5 minutes)

Purpose of the PI: audiences outside the research group will help the group achieve better research results.

The project: research process has reached the interpretation phase, key stage, but analytical results without interpretation says nothing - but: no one can know and think about everything, it also applies to scientists.

Demetriq revolves around the question: how does the development of labor market policies affect the chances of different socio-economic groups on the labor market and the risk of exclusion (which is considered to have a connection to the health inequalities between different social groups).

This is where you enter: actors with expertise with respect to the labor market were invited, who can contribute with different views than a social medicine research group

For peer review only - http://bmjopen.bmj.com/site/about/guidelines.xhtml

has - it is you who are [name of public group] [experts], but not only you, but also politicians, employers, unions, social insurance office, unemployment agency => to get the "full picture" to broaden the perspective, see more of the analysis results to achieve a better, more reliable interpretation

Use of mp3 player: ok if I start audio recording (capture as much info as possible)

Discussion Rules:

Remember (mp3 player):

 To talk without interruption + to talk one at a time

 To describe the piece of data you refer to with words

 Questions always welcome

Consent:

voluntary – stop at anytime

anonymous - no accounting at the individual level or by name (in the published articles and reports)

confidential - datasets only available to the research team

 Consent in presentation round

Presentation Round: name, position in the organization, consent (identifiable voice on "tape")

Introduction Demetriq - Expert (about 10-15 minutes)

Finish with questions

For peer review only - http://bmjopen.bmj.com/site/about/guidelines.xhtml

00: 20 to 00:50: Discussion part I: Employment

1a) presented results (20min): Empty timeline (1) + Employment Charts Sweden (2)

- If you think of the employment rate in Sweden, what events, regulations, developments in international / national level may have contributed to the development of employment (fill in the timeline)? It can be about events or processes regarding policies, but also other areas that you think are relevant (post-it discussion)
- What do you think of when you see the diagram of the employment rate in the various groups (notable parts of the graph) against the background of developments that you mentioned?
- Is it in line with your experience and knowledge?
- In what ways may the development of employment depend on issues you mentioned in the timeline (ideas for explanations, associations)?

1b) presented results (10 min): Employment Charts for SE, DK, NL, UK, compared

- What do you think when you see this European picture where all graphs show a negative trend for the target group?
- What do you think about Sweden's position in the European comparison?

00: 50-01: 10: Discussion part II: poverty risk

Presented results (20 min): Poverty Risk SE, DK, NL, UK

- What do you think of when you see the diagram of the risk of poverty in the different countries (notable parts of the diagram)?
- Is it in line with your experience and knowledge?

For peer review only - http://bmjopen.bmj.com/site/about/guidelines.xhtml

• Do you see a connection between poverty and issues you mentioned that you mentioned on the timeline? What then do you think causes the evolution of poverty risk?

01: 10-01: 30 Discussion part III: policy implications

Presented results (20 min): empty logic model

• What regulations and actions are hiding behind the concepts ALMP, flexibility, economic security? (still possible to add something)

• We would like to ask you to fill in the consequences you think that policies affecting economic security, flexibility and active labor market policies may have on employers and employees.

• We have come to this model (show), what do you think? Does it help with carrots / sticks?

01: 30-01: 45: Discussion part IV: flexicurity in Sweden

Presented results (15 min): 4-field diagram with countries DK, NL, UK, without SE

• Position Sweden in the table according to how flexible and secure you feel the Swedish labor market is, and state why you place Sweden where you see it in the table.

• Has there been development over the last 20 years that you can describe and what do you think the future development will look like?

What do these findings say on how to reduce inequalities in health in society? Have we selected the right target group (low edu LLI) and indicators data in that regard?

01: 45-02: 00: End of the Workshop: round up

• What do you take home of what we've talked about today (the most important)? Please summarize briefly.

For peer review only

For peer review only - http://bmjopen.bmj.com/site/about/guidelines.xhtml

• What should we take home as a form of research assignments, i.e. questions, focus, and the like for future research from your perspective as representatives of [name of public group]?

For peer review only

For peer review only - http://bmjopen.bmj.com/site/about/guidelines.xhtml

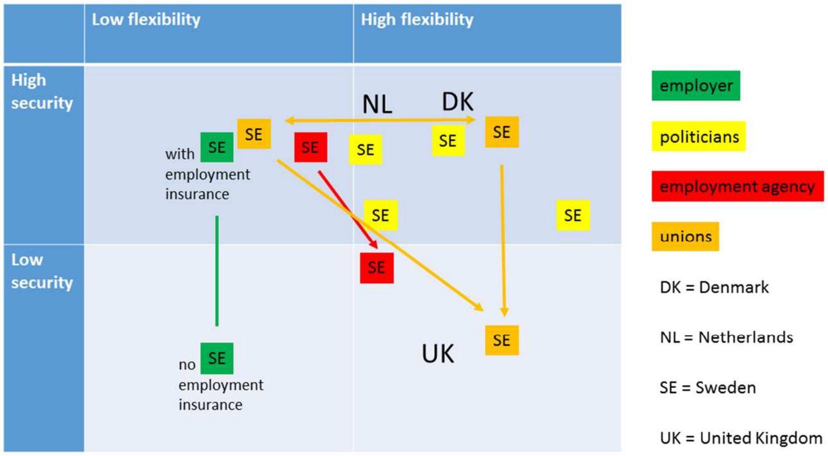

Fig 1: Matrix showing the different categorizations of Sweden's degree of Flexicurity by the 4 public groups

338x190mm (96 x 96 DPI)

BMJ Open

Involving the public in epidemiological public health research: a qualitative study of public and stakeholder involvement in evaluation of a population-wide natural policy experiment

Journal:	*BMJ Open*
Manuscript ID	bmjopen-2017-019805.R1
Article Type:	Research
Date Submitted by the Author:	18-Jan-2018
Complete List of Authors:	Anderson de Cuevas, Rachel; University of Liverpool, Public Health & Policy<br>Nylén, Lotta; Karolinska Institutet, Department of Public Health Sciences, Division of Social Medicine<br>Burström, Bo; Karolinska Institutet, Department of Public Health Sciences, Division of Social Medicine<br>Whitehead, Margaret; University of Liverpool, Public Health and Policy
**Primary Subject Heading**:	Public health
Secondary Subject Heading:	Research methods
Keywords:	social determinants health, health inequalities, public involvement, natural policy experiment, PUBLIC HEALTH, flexicurity

SCHOLARONE™
Manuscripts

For peer review only

Involving the public in epidemiological public health research: a qualitative study of public and stakeholder involvement in evaluation of a population-wide natural policy experiment

Authors:

Rachel Anderson de Cuevas[1], Lotta Nylén[2], Bo Burström[2*], Margaret Whitehead[1]

Affiliated institutions:

[1]Department of Public Health and Policy, University of Liverpool, Liverpool, Merseyside, United Kingdom; [2]Department of Public Health Sciences, Karolinska Institutet, Stockholm, Sweden.

*Corresponding Author: Bo Burström, Professor of Social Medicine, Division of Social Medicine, Dept. of Public Health Sciences, Karolinska Institute, Department of Public Health Sciences (PHS), K9, Social Medicin, 171 77 Stockholm, Sweden, Email: Bo.Burstrom@ki.se

Keywords: health inequalities; social determinants of health; Europe; public involvement; flexicurity.

Word count: 3995

For peer review only

Abstract

Background: Public involvement in research is considered good practice by European funders; however evidence of its research impact is sparse, particularly in relation to large-scale epidemiological research.

Objectives: to explore what difference public and stakeholder involvement makes to the interpretation of findings from an evaluation of a natural policy experiment to influence the wider social determinants of health: 'Flexicurity'.

Setting: Stockholm County, Sweden.

Participants: Members of the public from different occupational groups represented by blue-collar and white-collar trade union representatives. Also, members of three stake-holder groups: the Swedish national employment agency; an employers' association and politicians sitting on a national labour market committee. Total: 17 participants.

Methods: Qualitative study of process and outcomes of public and stakeholder participation in four focussed workshops on the interpretation of initial findings from the Flexicurity evaluation.

Outcome measures: New insights from participants benefitting the interpretation of our research findings or conceptualisation of future research.

Results: Participants sensed more drastic and nuanced change in the Swedish welfare system over recent decades than was evident from our literature reviews and policy analysis. They also elaborated hidden developments in the Swedish labour market which were increasingly leading to "insiders" and "outsiders", with differing experiences and consequences for financial and job security. Their explanation of the differential effects of the various collective agreements for different occupational groups was new and raised

For peer review only

further potential research questions. Their first-hand experience provided new insights into how changes to the social protection system were contributing to the increasing trends in poverty among unemployed people with limiting long-standing illness. The politicians provided further reasoning behind some of the policy changes, and their intended and unintended consequences. These insights fed into subsequent reporting of the Flexicurity evaluation results, as well as the conceptualisation of new research that could be pursued in a future programme.

Strengths and limitations of this study

- To our knowledge, this is the first report of the impact of involving the public and stakeholders in population-wide epidemiological studies, which require interpretation of national quantitative datasets.
- It provides a reasoned solution to the challenge of developing the most appropriate public and stakeholder involvement for quantitative public health research on the wider social determinants of health.
- A limitation is that the public and stakeholders were involved in one discrete phase of the research process, rather than throughout.
- This limitation is being addressed by the continued involvement of participants in the conceptualisation and planning of future research from their ideas coming out of the current study.

For peer review only

Introduction and background

Public and patient involvement in health and social research is regarded by funding bodies and policy makers as an integral part of good practice and democratization of the research process (1). There is growing recognition that lay people have valuable expertise that should inform the research process, as well as having the right to be more actively involved in the design and outputs of research that is about and for them. Increasingly, research funding bodies also require researchers to demonstrate the value of patient and public involvement by evidencing its impact (2).

There is now a substantial body of evidence on good practice in involving patients in research to improve the services that they need (3-5) and a growing literature on involving the wider public in community-based prevention and health promotion research, for example engaging residents of areas in which neighbourhood initiatives are based (6, 7). There are few studies, however, about the involvement of the public in population-wide epidemiological studies, including the evaluation of nation-wide policies to influence the wider social determinants of health (such as employment and poverty). Arguably, one of the main reasons for this dearth of public involvement studies at the population level is the many additional challenges that public health research of this nature throws up. Not least among these challenges is the selection of the most appropriate representatives from amongst “the general public” and the question of where in the process, and in what form, that involvement would be both effective and acceptable to those involved, given the highly technical nature of the statistical analysis of epidemiological datasets.

In this paper, we aim to present the findings of our attempts to tackle some of these challenges, by incorporating public and stakeholder involvement in the evaluation of the

For peer review only

impact on health inequalities of a natural policy experiment in labour market 'flexicurity'. We explain what we did and why, and how that involvement benefitted the interpretation of our research findings and conceptualisation of future research.

'Flexicurity' and the employment of people with a disability or chronic illness

There is a general rise across Europe in the numbers of disabled and chronically ill people of working age that are not in employment. This is a matter of growing concern for governments due to its adverse consequences for public health and potential to exacerbate health inequalities, as well as its effect on increasing social security expenditure (8). Ageing populations and a raised retirement age in several countries mean that the proportion of the working age population leaving the labour force due to ill-health is expected to rise further (8, 9).

In Sweden, the proportion of the population receiving sickness or activity compensation has trebled in the last 30 years (10). The combined cost of sick leave and disability pensions equates to approximately 4% of Swedish GDP and nearly 8% of the working age population receive disability pensions (8). The situation in Denmark and Norway is similar (8 and 10%, respectively), while in the UK, more than 2.6 million people receive incapacity-related benefits, accounting for a quarter of the social security budget and 1.5% of GDP (11).

Being outside the labour market is likely to add to the social and economic exclusion of people with a disability or illness, as income, opportunities to participate fully in society and quality of life decrease (8, 9). People in poor health have lower employment prospects than the general population and these prospects diminish in line with declining socioeconomic position (12, 13). The social gradient in the employment of people with disabilities may serve to widen health inequalities, since the more disadvantaged who already have a higher

For peer review only

prevalence of ill health will be further disadvantaged by the negative effects of unemployment (9).

European countries have undertaken a range of labour market initiatives which may affect the ability of chronically ill and disabled people to get and keep a job (8). Population-wide policies centre around two axes: how flexible the labour market is (the degree of regulation and employment rights) and how much financial security the welfare system offers to disabled people who are out of work. These policies have generated considerable debate about their possible impact on the employment opportunities of chronically ill and disabled people in different socio-economic groups.

Increased flexibility of employment conditions is considered to have contributed to higher levels of job insecurity and to have disproportionately affected those who find themselves in the most vulnerable positions in the labour force (including women, the low skilled and people with disabilities) (14). Since labour market deregulation is considered an essential strategy to redress the economic downturn, 'flexicurity' has been proposed as an alternative; compensating for increased flexibility with provisions of security (10). Flexicurity policies aim to integrate flexible labour market with strong financial security and active labour market policies. (Denmark and the Netherlands are often cited as exemplars of flexicurity in its most developed form). As part of the DEMETRIQ project funded by an FP7 EU grant, we investigated the impact of flexicurity policies on social inequalities in the chances of being in employment for people with and without limiting longstanding illness in European countries. To do this, we exploited, for research purposes, the contrasts in flexibility and financial security found in exemplar countries, which provided the necessary

For peer review only

variation for a natural policy experiment (see for example (15)). The case study in public involvement reported here was conducted as a component of the Swedish country study.

Study design and methods

The qualitative study aimed to explore how public and stakeholder involvement could be incorporated effectively into the evaluation of the health inequalities impact of flexicurity policies and how that affected (if at all) the interpretation of findings and implications for future research.

Study participants: The first question to be resolved is "who is 'the public?' in the context of the evaluation of population-wide employment policies. As the umbrella project was focused on inequalities in health, the EU commissioning brief identified an additional need for "further research on involving population groups with least power and resources, who are intended to be the ultimate beneficiaries of the research" (16). With this proviso and the population level context in mind, we made the following selection decisions:

1. The ultimate beneficiaries of our flexicurity research were people in the most vulnerable positions in the labour market, specifically groups with low skills/low education. Those with low education and disabilities/long-standing illness were doubly disadvantaged.

2. Participants for this explorative qualitative study would be drawn from one European country: Sweden, from where the overall leadership of the flexicuirty evaluation was based, and which was representative of a recognised type of flexibility and security policy system (i.e. low flexibility and high security).

For peer review only

3. We needed to avoid 'tokenism', whereby a few low skilled workers or unemployed people with disabilities were selected at random, without consideration of the nature of the research data they would be engaging with. Following discussions with PPI experts and deep knowledge of the health inequalities literature, we decided that it would be more appropriate to involve trade union representatives from blue-collar and white collar unions as members of the relevant public. They were workers themselves, but also had the added experience of how their fellow workers in different socio-economic circumstances and with different health conditions fared under the various national policies. They were also familiar with considering labour market trends at the population level. In addition, we decided to invite participation from representatives of other relevant public bodies, who could be more accurately termed 'stakeholder groups' rather than 'members of the public'. These stakeholders were members of: the Swedish employers' association (as the Swedish labour market is reliant to a greater extent than elsewhere on collective agreements between trade unions and employers); the Swedish national employment agency (as the public body that implements policies to get unemployed people back to work, and has a special remit for disabled and chronically ill workers); and politicians from the ruling coalition and opposition parties (who sat on a national labour market committee).

Study design and conduct: The study component reported here comprised qualitative research involving members of the public (as defined above) and stakeholder groups in the interpretation of research findings and reflections on future research. Four focused workshops with participants were held using a process developed by Petticrew and

For peer review only

Whitehead (17, 18). Trade union and stakeholder participants were identified from the research group's existing research users' network, using a snowballing technique. Potential participants were sent invitation letters, an outline of the research project and the objectives of the public involvement activity. Interested participants were given further information about the research and engagement activity through telephone conversations, face-to-face meetings and written correspondence.

The planning, process and outcomes of the focused workshops were recorded and evidenced in parallel to the activity. Preliminary research results were presented to participants in graphical form during focused workshops, and then participants' perspectives on selected research findings elicited.

Two researchers with expertise in qualitative research methods and public engagement, who were independent from the primary study, designed and led the public involvement activity and conducted the analysis. An independent moderator facilitated the workshops.

Description of focused workshop activity

Selected findings were presented at four 2-hour focused workshops held separately with each public and stakeholder group at their workplace during June and August 2014. Two to six participants attended each session, with a total of 17 participants. Findings were presented in diagrammatic form (as described in Box 1). One researcher moderated the discussion and 1 or 2 members of the core research team presented the findings. A standardised topic guide was followed (Appendix 1), with additional questions tailored to suit each group's expertise and interest. Focused workshops were conducted and transcribed in Swedish and selected quotations translated into English.

For peer review only

Box 1: Description of material presented at the focused workshops

Participants were presented with a graph showing trends in Swedish employment rates of people with a limiting longstanding illness (LLI) and healthy people stratified by education level from 1990 to 2011, constructed from the Swedish Survey on Living Conditions and a blank timeline for the same period. Participants were then invited to chart important events or trends during this period on the timeline and to interpret developments in the employment of people with LLI and healthy people in the light of these events. Participants then compared graphs presenting differences in the employment rate of those with and without LLI and with high and low educational levels in Sweden, Denmark, the Netherlands and the United Kingdom for the period 2005 – 2011 from EU SILC data (European Union Statistics of Income and Living Conditions, EUROSTAT). They commented and reflected on the situation in Sweden compared with the other 3 countries. The differential rate of 'poverty risk' (<60 per cent of median income) among non-employed/employed people with LLI and employed healthy people was also presented in graphical form (EU SILC) and participants were asked to interpret changes in poverty risk in light of the key socio-political or economic events they had charted on the timeline. Lastly, participants considered the categorisation of the UK, Netherlands and Denmark in relation to their degree of employment protection and economic security (Flexicurity) in a 2x2 matrix and reflected where to best position Sweden and the trajectory of the country's position over time.

Analytic framework

Full transcripts of the digital recordings of the focused workshops were coded and categorised, employing the principles of content analysis, for the dataset as a whole and

For peer review only - http://bmjopen.bmj.com/site/about/guidelines.xhtml

separately for each workshop (19, 20). Thematic analysis of the moderator's notes and the transcripts drew out key themes from each workshop to form a summary of themes across the workshops and to compare the insights of the 4 different public and stakeholder groups between workshops (21). Transcription and analysis were undertaken alongside the focused workshop activities as part of an iterative process which informed each phase of data collection (22). The Swedish and UK research teams as a whole then reflected on the findings from the focussed workshops and considered how these informed the researchers' original interpretation of their epidemiological data or generated new research questions for future research.

Research ethics

Ethical clearance was not required under Swedish law on ethical review (refer to Lag 2003:460), as the information collected was not considered to constitute sensitive personal information (§3 in the Law). Stakeholders participated as collective members of their employing institution, not as individuals or the subjects of the research. All gave informed consent to being interviewed and for the interviews to be recorded and transcribed.

For peer review only

Results

Participants were all employed women and men aged 40 to 60 years (17 participants). All groups commented on the decline in the employment rate of low educated people with LLI over time and offered a range of explanations for this development (Table 1).

For peer review only

Table 1. Summary of explanations for epidemiological results from participants of focussed workshops.

What explains lower employment rate in target group?	Which policy changes have occurred?	What explains increasing rates of relative poverty among non-employed?	Has Sweden moved to flexicurity?
Globalisation: higher demands on workers, higher education requirements.<br>Economic recessions early 1990s, early 2000 and 2008. (TU, E, EA, P)	Fighting inflation instead of unemployment, to be competitive (TU).<br>De-regulation of many sectors (e.g. transport, construction) (TU)	Increasing market incomes, tax reductions. (P)<br>Stagnant levels of unemployment and social assistance benefits. (P)	Varies between individuals – if you are part of unemployment insurance and collective agreements, you are secure; otherwise not. Labour market not flexible (E).
Changing labour market:<br>New complex technology – increased demand on workers.<br>Reduced demand for unqualified workers (TU, E, EA, P)	Less emphasis on vocational training and life-long learning (TU, EA)	Security has moved to other arenas – collective agreements and individual choices more important (TU, E)	Flexibility for employers has increased. Security has become differentiated – not general. Depends on where you are in the system (TU)
Changing composition of group with low education and LLI – more low educated immigrants with refugee background.<br>(E, EA)	Social security reform in 2008 - reduced duration of sickness absence, restrictions on disability pension (TU, E, EA, P).	Fewer eligible for disability pension, fewer covered by unemployment insurance (EA)	Decreased economic security, not increased flexibility for the employer. Varies between different employers and white/blue collar trade unions, depends on which sector. (EA)
Increasing levels of mental ill health – more difficult to find jobs, lock-in effects. Focus on individual's efforts. (EA)	Reduced economic security to incentivize work (EA)	Reduced economic security to incentivize work (EA)	Politicians divided on the issue – most agree that flexibility is high; economic security is also considered high, but social democrats and green party think security has been lowered (P).
Social insurance reform shifted large groups from social insurance to unemployment (TU, EA)	Security has moved to other arenas – collective agreements and individual choices more important (TU, E)	Increased unemployment insurance fee – lower participation (TU,P)	
Target group less likely to be employed (E)	Extended period for employers to pay sickness benefits (E).	More unemployed rely on social assistance (EA, TU)	

Key: E = employers; P = politicians; TU = trade unions; EA = employment agency.

For peer review only

Explanations for the lower employment rate of low educated people with LLI in Sweden

Participants put forward the economic recessions of the early 1990s, early 2000s and 2008, as explanations for the low employment rate of low educated people with LLI in Sweden: *"That's how it is. We know that in every recession […] they fall further and further down. They are last in line."* (Trade unionist).

Many considered economic cycles to have had a greater influence on employment rates than national policies. The employer's association and some of the politicians emphasized that the pressures of global competition had led to outsourcing of low-skilled production tasks abroad, placed greater exigencies on workers in employment and removed demand for low-skilled workers with health problems. As one politician put it: *"… we have a labour market which does not have room for these people; as [was the case] … in the 1990s …"* (politician)

The 2008 reform of Swedish social insurance limited the time allowed for sickness absence and shifted a large group of people from social insurance to unemployment benefits. This was noted by the employment agency: *"those expelled from the insurance… the first batch … - [I] think it was 4th January 2010 - … 14,000 people suddenly came to the employment agency."*

The composition and size of the low educated group with LLI was also seen to have changed. The group had become smaller, as a result of the rise in the educational level of the general population and included a higher proportion of immigrants and people with mental ill-health. This was seen as another explanation for the group's lower employment rate: *"A large proportion of recent immigrants are very low educated or illiterate and of*

For peer review only

course they have greater difficulties [in securing employment] …" (Employment agency).

Another employment agency employee said that it was harder for people with functional disabilities resulting from mental illness to find jobs: *"… it is easier to adjust physically … but if you have ADHD and forget your appointment time …"*

A change in work place attitude towards people with disabilities was also mentioned as a factor contributing to the difficulty for low educated people with LLI to find employment: *"The climate has sort of become tougher"* (*Employment agency*).

What are the key policy changes in Sweden that have influenced the employment of people with low education and LLI from 1990-2010?

Trade unionists suggested that changes in economic policy underpinned some of the trends in the employment rate of low educated people with activity limitations due to health: *"I believe that the higher structural unemployment that results from prioritizing low inflation over low unemployment enables employers to pick and choose. And then they pick the high educated … rather than the low educated."* Deregulation of many different sectors was also mentioned: *"[The] taxi [industry] in Sweden is the most de-regulated in the world."*

Trade unionists felt that that there had previously been much more emphasis on providing vocational training and continuing education for people with LLI who were not employed, whereas today individuals – particularly blue collar workers – are simply urged to look for and obtain a job: *"… you would think our government could step in and make sure these groups get at least an equal chance and support blue collar workers."*

For peer review only

Some participants, including trade union representatives, described how universal social security systems had been eroded over time. Unemployment insurance contributions had increased substantially, resulting in many employees leaving the scheme. Unemployment benefit levels had stagnated or been lowered and social assistance benefits had plateaued. In parallel with the erosion of universal social security systems, collective agreements between employers and employees had changed, partly to compensate for this phenomenon. As one trade unionist put it:

"Security has moved to other arenas, because, for instance, if you don't increase the unemployment insurance, it has moved to other types of insurance,, that's how it is, then parts of the security system have moved."

The "other arenas" included collective agreements. During the discussion, the trade unionists explained that up to the 1990s, Sweden used to have national policies and security systems which would cater for all in the same way, in case of unemployment or redundancy. The active labour market policies (e.g. re-training etc.) benefitted those with lower education. The unemployment insurance was quite generous and adequate (at least for those with low/normal incomes). However, over the years, as the benefit levels were not increased and active labour market policies were reduced, the different unions tried instead to supplement with their own insurances, linked to specific collective agreements. The white collar unions with lower unemployment risk could negotiate better terms than blue collar unions – and as a result the differential increased further, and became dependent on the unemployment risk. Those in the most marginal positions on the labour market (and not subscribing to the unemployment insurance) were even worst off.

For peer review only - http://bmjopen.bmj.com/site/about/guidelines.xhtml

Employers mentioned another policy influence on people with LLI; an extension to the period of time an employer is responsible for paying sickness benefits for an absent employee, thereby increasing the cost to the employer. This policy change may have been detrimental to the employment chances of persons with LLI. Other reforms to social security regulations, including the aforementioned restriction to the duration of sickness absence and also to eligibility for disability pension, were considered by many participants to be important policy changes aimed at incentivising work: *"It's tougher to be an outsider [to the labour market] today than [it was] 6 years ago … This is partly due to a political desire to strengthen the incentive to work"* (Employment agency).

What explains increasing rates of relative poverty among non-employed persons with limiting longstanding illness in different European countries?

Workshop participants were shown results comparing the prevalence of relative poverty among employed and non-employed people with limiting longstanding illness in Sweden, Denmark, the Netherlands and the United Kingdom, for the years 2005 and 2010, respectively. In Sweden, the poverty rate had nearly doubled from 2005 to 2010 albeit from a very low starting point, whereas increases in the other countries, which started with higher poverty rates in 2005, were smaller. Some politicians questioned the validity of the measure "relative poverty" (defined as having an income below 60 percent of the median national income): *"… it describes an increasing income difference in society, but it says nothing about whether poverty has increased. These are two completely different things."*

Other participants attributed the increasing rates of relative poverty directly to national policies: *"… it has been a deliberate policy … to increase the differences in income according*

For peer review only - http://bmjopen.bmj.com/site/about/guidelines.xhtml

to whether you work or do not work, … so you notice the change if you start working" (Employment agency).

The social insurance reform of 2008 had excluded many non-employed people with LLI from sickness benefit, yet many were not on the unemployment register. The issue was how they made their living: *"… they are somehow supported by relatives … The group with mental problems … seek social assistance more than others"* (Employment agency).

The increase in unemployment insurance contributions (due to a change in government policy in 2007) led to many employees leaving the insurance and was also put forward as an explanation for rising rates of relative poverty among this population group: *"… there are many unemployed people who are not part of the unemployment insurance scheme at all, and who don't get anything …"* (Employment agency).

Has Sweden moved to flexicurity?

Workshop participants were asked whether they considered that the policy changes that had taken place from 1990 to 2010 had moved Sweden to qualify as a 'flexicure' country and were asked to place Sweden in a matrix of high/low flexibility and high/low economic security (Figure 1). (Denmark, the Netherlands and the UK were positioned by the researchers prior to the workshops in line with the evidence from the policy literature). Opinions were divided. One trade unionist commented: *"The whole of [Swedish] working life has slipped towards the UK [position, with high flexibility/deregulation and low economic security] …"* Another trade unionist also thought that flexibility had increased: *"In Sweden, we have perhaps the highest flexibility out of many countries, to fire people … It is incredibly liberal … We belong to high flexibility."*.

For peer review only

For peer review only - http://bmjopen.bmj.com/site/about/guidelines.xhtml

Employers, on the other hand, considered that flexibility was low, but that the degree of economic security differed according to whether or not an employee was part of unemployment insurance and covered by collective agreements. For those who did not belong to these schemes, security was lower than before: *"… the universal welfare system has been hollowed out since the 1990s"* (Employer).

Trade unionists and employers referred to a two-tier system, in which the situation for 'insiders' to the labour market - those who have had a job for some time, who are part of the unemployment insurance scheme and are covered by collective agreements - was much better than for those outside these arrangements. Most politicians commented that Sweden had high flexibility and high security, having moved from lower flexibility and even higher economic security. One politician spoke of: *"… a divided labour market … There are those who have security and those who do not …"*

For peer review only

Fig 1: Matrix showing the different categorizations of Sweden's degree of Flexicurity by the 4 public groups

For peer review only

Impact of this involvement on researchers' interpretation of findings

Subsequent to the focused workshops with public and stakeholder representatives, the wider research team comprising Swedish and UK members held reflective sessions to consider if, and how, the involvement activity had influenced their initial interpretation of findings or highlighted gaps in the evidence base.

Four main insights were identified, triggered by the public and stakeholder involvement, which deepened our understanding of our findings and influenced subsequent publications. First was the extent to which people experiencing the labour market and welfare reforms first hand (at 'the coal face' so to speak) sensed more drastic and nuanced change in the Swedish welfare system over the past two decades than was evident from the academic literature reviews and policy analyses. The exercise resulting in figure1 highlighted this point visually.

Second was the introduction of the concept of "insiders" and "outsiders" in the labour market, emerging from the workshops, particularly from discussions among trade unionists and employers. This highlighted the differing experiences and consequences for employees depending not just on SES or simple blue-collar/white collar dychotomy, but also on the length of time employees had had a job and whether or not they were part of the whole infrastructure which provided financial and job security. This concept fed into the generation of hypotheses to be tested in secondary data analysis, the results of which we subsequently published in a peer-reviewed paper (15).

Third was the possibility of differential effect of collective agreements for different occupational groups. It was the deep understanding by unions and employers of how collective agreements had operated in practice over the years that offered the researchers

For peer review only - http://bmjopen.bmj.com/site/about/guidelines.xhtml

fresh insights into how groups that were already disadvantaged could be hardest hit by poorer, or no, collective agreements. This led to the development of a research proposal on the role of collective agreements in contributing to differential impacts of labour market policies, which was submitted by the research team to a Swedish national research funder: FORTE.

Fourth, triangulation of perspectives from the different public and stakeholder groups gave a much fuller picture of what was happening as a result of the interactions of people with the system. The trade unions and employers raised issues about the consequences of collective agreements as detailed above. The employment agency described from first-hand experience the impact of changing policies on the lives and finances of unemployed people, particularly those who were “outsiders”. The politicians understood, from their own experience of legislating the stated and real reasons for changes in policies and the intended and unintended consequences.

Discussion

This is a rare example of a study exploring what difference public and stakeholder involvement makes to an evaluation of a natural policy experiment to influence the wider social determinants of health. Furthermore, it was conducted in a European country outside the UK, where the concept of lay involvement is less developed. To our knowledge, this is also the first report of involving the public and stakeholders in population-wide epidemiological studies, which require interpretation of national quantitative datasets (4).

Reservations have been expressed elsewhere about the difficulties of involving the public in research that is highly technical (23). Added to that was the population-wide nature of the

For peer review only - http://bmjopen.bmj.com/site/about/guidelines.xhtml

policies being evaluated. Our decisions on *who* to involve and *how* were therefore critical to the whole research endeavour reported here. Our final selection of trade union representatives from blue-collar and white-collar unions as the most appropriate members of the relevant public, coupled with the decision to involve stakeholders from the employers' organisation, public employment office and politicians serving on employment policy committees, proved both feasible and productive. Participants in the focussed workshops demonstrated a high level of lay expertise and a capacity to engage with the intricacies of policy developments, changes to the economy, the country's demographic profile and global market forces, from both a practical and theoretical perspective. They expressed appreciation of the way the study findings were presented to them for comment – as "*big trends over time*" – rather than facing them, for example, with the technicalities of the epidemiological analysis. There is continuing debate about how to facilitate lay involvement in research and at what point would be most appropriate (24-26). The focussed workshops reported here provide an example of what helped public and stakeholder involvement in the context of an evaluation of a population-wide natural policy experiment.

The study concluded that this public and stakeholder involvement benefited the interpretation of the research findings and conceptualisation of future research in a number of ways. Participants sensed more drastic and nuanced change in the Swedish welfare system over recent decades than was evident from our literature reviews and policy analysis. They also elaborated hidden developments in the Swedish labour market which were increasingly leading to "insiders" and "outsiders", with differing experiences and consequences for financial and job security. The explanation by the trade unionists and

For peer review only - http://bmjopen.bmj.com/site/about/guidelines.xhtml

employers of the differential effects of the various collective agreements for different occupational groups was new and had not been a focus in the studies performed by the researchers. The first-hand experience of the employment agency provided new insights into how changes to the social protection system were contributing to the increasing trends in poverty among unemployed people with limiting long-standing illness. The politicians provided further reasoning behind some of the policy changes, and their intended and unintended consequences. These insights fed into our subsequent reporting of the results of the Flexicurity evaluation (15, 27, 28), as well as the conceptualisation of new research that could be pursued in a future programme (see below).

Limitations

The main limitation was that public and stakeholder involvement was limited to one discrete phase of the research process: the interpretation of initial findings. There is some evidence to suggest that involving the public throughout the research process (as well as long-term involvement and continuity of membership beyond a single research project) enhances impact (4, 29, 30). While we judged involvement in this discrete phase to be the most practical and logical starting point faced with the specific population-wide policy evaluation of flexicurity, it became clear during the focussed workshops that this could also be a way of building a continued relationship for future research. The research ideas coming out of the workshops and the willingness on the part of participants to be involved in a future programme, laid the groundwork for involvement in future research from the very beginning, including the conceptualisation of the research questions.

A higher-level limitation is that research on public and stakeholder involvement is inherently context-specific, as the impact of involvement is likely to depend on many factors, including

For peer review only - http://bmjopen.bmj.com/site/about/guidelines.xhtml

the topic being studied, the public groups being represented, the perspective of the researchers, and the extent of involvement. Producing generalizable evidence in the area of public involvement is inherently difficult. This study was conducted in the context of Sweden and its labour market and social protection system. Mindful of this, we tried to tailor the public and stakeholder involvement initiatives to take account of the country context (Sweden), the topic (policies around Flexicurity) and the nature of the research in which involvement is being encouraged (population-wide epidemiological studies). In this type of research, understanding and taking account of the context is essential if the findings are to be of value to research users in Sweden and elsewhere.

Input into the conceptualization stage of future research

The public and stakeholder involvement, although only at the interpretation of findings stage for the current research, gave rise to new research questions to address in a future research programme. Could it therefore be a way of introducing public involvement right from the conceptualization stage in future population-wide epidemiological studies of this nature? Ideas for future research that came out of the public and stakeholder involvement included the following.

First was the question of how the changing composition of, and the increasing prevalence of neuropsychiatric diseases in, the group of low educated people with LLI might affect the employment chances of the group. This is an interesting issue to pursue in view of the increasing demand for social skills on the labour market, also in lower skilled jobs.

Another idea for future studies came from the unionists' statement of differential conditions associated with different collective agreements, that "security has moved to other arenas", i.e. from the national level to the level of specific agreements negotiated by

For peer review only - http://bmjopen.bmj.com/site/about/guidelines.xhtml

individual unions. Here, detailed assessment of conditions pertaining to different groups of manual workers, compared to conditions among higher non-manual employees, could help identify differential impacts and trajectories of different groups in the event of unemployment. This idea has been worked up, jointly with participants, into a research proposal, which was submitted to a Swedish national funder, FORTE.

Thirdly, the employment agency offered the use of their more detailed data to study the employment and economic situation of specific groups, such as people with psychological illnesses, whose employment prospects and economic situation may be poorer than among those with physical illnesses, not least following recent restrictions in social insurance policies.

Hence, early contacts with the public and with stakeholders, who day to day face the concrete impact of new policies or other changes on the labour market, may be very pertinent to specifying research questions addressing the impact of such real life changes. Increased involvement and dialogue with the public and with stakeholders in the identification and framing of specific research questions may therefore contribute both to increased scientific rigour, as well as societal relevance of policy oriented research.

For peer review only

Conclusion

This study shows that it is possible to incorporate public and stakeholder involvement in a meaningful way in epidemiological public health research. The insights and reflections the public and stakeholders offered added depth and subtlety to the researchers' interpretation of the research findings and highlighted the issues perceived to be of most public relevance. In addition, participants raised questions that could not be answered by the current study, which stimulated ideas for future research and contributed to specifying highly relevant research questions. Early engagement with the public and with stakeholders may therefore contribute to improve both the scientific rigour and the relevance of policy oriented research.

For peer review only

For peer review only - http://bmjopen.bmj.com/site/about/guidelines.xhtml

Contributorship statement:

MMW and BB conceived the research study. LN and RMAdC designed and led primary data collection and analysed the data. RMAdC and BB wrote the first draft and together with MMW and LN interpreted the data. All authors contributed to the final draft.

Competing interests:

The authors have no conflicts of interest to declare.

Funding:

This research was funded by an European Commission FP7 grant DEMETRIQ (developing methodologies to reduce inequalities in the determinants of health) (Grant Reference: 278511), http://www.demetriq.eu/. The funders had no role in study design, data collection and analysis, decision to publish, or preparation of the manuscript. The authors of this paper alone are responsible for the views expressed in this publication which do not necessarily represent the decisions or policies of the EU or their institutions.

Data sharing statement:

Transcripts of the focussed discussions, field notes and observations are in Swedish. To protect the anonymity of the public and stakeholder participants, we cannot share the primary qualitative data with third parties.

For peer review only - http://bmjopen.bmj.com/site/about/guidelines.xhtml

References

1. Boote J, Baird W, Beecroft C. Public involvement at the design stage of primary health research: a narrative review of case examples. Health policy (Amsterdam, Netherlands). 2010;95(1):10-23.
2. Snape D, Kirkham J, Britten N, Froggatt K, Gradinger F, Lobban F, et al. Exploring perceived barriers, drivers, impacts and the need for evaluation of public involvement in health and social care research: a modified Delphi study. BMJ open. 2014;4(6):e004943.
3. Brett J, Staniszewska S, Mockford C, Herron-Marx S, Hughes J, Tysall C, et al. Mapping the impact of patient and public involvement on health and social care research: a systematic review. Health expectations. 2012;17(5):637-50.
4. Staley K. Exploring impact: Public involvement in NHS, public health and social care research. Eastleigh: INVOLVE, 2009.
5. Ocloo J, Matthews R. From tokenism to empowerment: progressing patient and public involvement in healthcare improvement. BMJ quality & safety. 2016;25(8):626-32.
6. O'Mara-Eves A, Brunton G, McDaid D, Oliver S, Kavanagh J, Jamal F, et al. Community engagement to reduce inequalities in health: a systematic review, meta-analysis and economic analysis. Public Health Res. 2013;1(4).
7. GoWell. GoWell Community Engagement: GoWell Glasgow Community Health and Wellbeing: Research and Learning Programme 2018 [updated 2018. Available from: http://www.gowellonline.com/about/components/community_engagement.
8. Whitehead M. Disability and employment: lessons from natural policy experiments. European journal of public health. 2010;20(4):371-3.
9. Clayton S, Barr B, Nylen L, Burstrom B, Thielen K, Diderichsen F, et al. Effectiveness of return-to-work interventions for disabled people: a systematic review of government initiatives focused on changing the behaviour of employers. European journal of public health. 2012;22(3):434-9.
10. Effective return to work. Reforms. Stockholm: Government Offices of Sweden, 2008.
11. Clayton S, Bambra C, Gosling R, Povall S, Misso K, Whitehead M. Assembling the evidence jigsaw: insights from a systematic review of UK studies of individual-focused return to work initiatives for disabled and long-term ill people. BMC public health. 2011;11:170.
12. Burstrom B, Holland P, Diderichsen F, Whitehead M. Winners and losers in flexible labor markets: the fate of women with chronic illness in contrasting policy environments--Sweden and Britain. Int J Health Serv. 2003;33(2):199-217.
13. Burstrom B, Whitehead M, Lindholm C, Diderichsen F. Inequality in the social consequences of illness: how well do people with long-term illness fare in the British and Swedish labor markets? Int J Health Serv. 2000;30(3):435-51.
14. Afzal Z, Muntaner C, Chung H, Mahmood Q, Ng E, Schrecker T. Complementarities or contradictions? Scoping the health dimensions of "flexicurity" labor market policies. Int J Health Serv. 2013;43(3):473-82.
15. McAllister A, Nylen L, Backhans M, Boye K, Thielen K, Whitehead M, et al. Do 'Flexicurity' Policies Work for People With Low Education and Health Problems? A Comparison of Labour Market Policies and Employment Rates in Denmark, The Netherlands, Sweden, and the United Kingdom 1990-2010. Int J Health Serv. 2015;45(4):679-705.
16. EC. FP7-HEALTH-2011-two-stage: Cooperation programme: European Commission; 2010 [updated 20-07-2010. Available from: https://ec.europa.eu/research/participants/portal/desktop/en/opportunities/fp7/calls/fp7-health-2011-two-stage.html.
17. Whitehead M, Petticrew M, Graham H, Macintyre SJ, Bambra C, Egan M. Evidence for public health policy on inequalities: 2: assembling the evidence jigsaw. Journal of epidemiology and community health. 2004;58(10):817-21.

For peer review only - http://bmjopen.bmj.com/site/about/guidelines.xhtml

18. Petticrew M, Whitehead M, Macintyre SJ, Graham H, Egan M. Evidence for public health policy on inequalities: 1: the reality according to policymakers. Journal of epidemiology and community health. 2004;58(10):811-6.
19. Robson C. Real World Research. 2nd edition ed. Oxford: Blackwell; 2002.
20. Graneheim UH, Lundman B. Qualitative content analysis in nursing research: concept, procedures and measures to achieve trustworthiness. Nurse Education Today. 2004;24(105-112).
21. Braun V, Clarke V. Using thematic analysis in psychology. Qualitative research in psychology. 2006;3(2):77-101.
22. Ritchie J, Lewis J, editors. Qualitative research practice: a guide for social science students and researchers. London: Sage Publications Limited; 2003.
23. Barber R, Boote JD, Parry GD, Cooper CL, Yeeles P, Cook S. Can the impact of public involvement on research be evaluated? A mixed methods study. Health expectations. 2012;15(3):229-41.
24. The Public Involvement Impact Assessment Framework Guidance. . Universities of Lancaster, Liverpool and Exeter, 2014.
25. PiiAF. Public involvement impact Assessment Framework (PiiAF) Resources: Draft standards for good practice in public involvement in research. MRC, 2014.
26. Gradinger F, Britten N, Wyatt K, Froggatt K, Gibson A, Jacoby A, et al. Values associated with public involvement in health and social care research: a narrative review. Health Expectations. 2013.
27. Backhans MC, Mosedale S, Bruce D, Whitehead M, Burstrom B. What is the impact of flexicurity on the chances of entry into employment for people with low education and activity limitations due to health problems? A comparison of 21 European countries using Qualitative Comparative Analysis (QCA). BMC public health. 2016;16(1):842.
28. Burström B, et al. Demetriq Project Report to the European Union. 2015.
29. Staniszewska S. Patient and public involvement in health services and health research: A brief overview of evidence, policy and activity. Journal of Research in Nursing. 2009;14(4):295-8.
30. PiiAF. Database – Methods and tools to assess impacts: Signposting resource to published case examples of methods and tools. PiiAF, 2013 Contract No.: 1st May 2014.

For peer review only

For peer review only - http://bmjopen.bmj.com/site/about/guidelines.xhtml

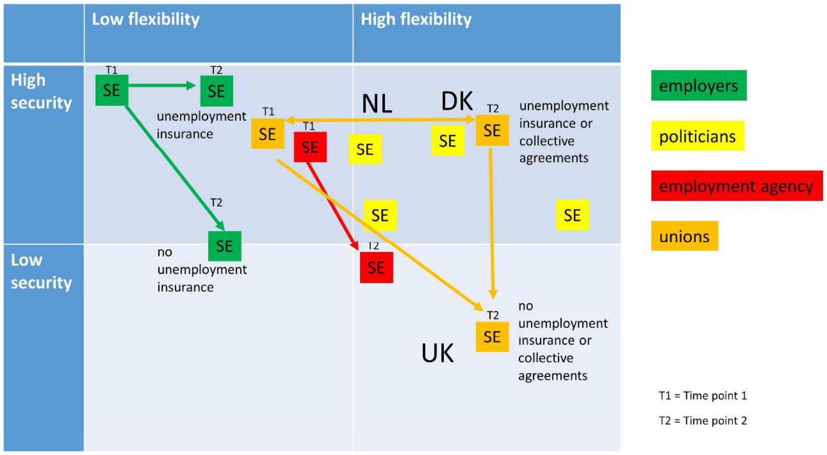

Fig 1: Matrix showing the different categorizations of Sweden's degree of Flexicurity by the 4 public groups

338x190mm (300 x 300 DPI)

S1 Appendix 1: Focused Workshop standard topic guide

Public

WS-leader Expert (introduce Demetriq + results), Moderator (questions, discussion)

Dual audio recording (mp3 + smartphone)

Demetriq = Developing methodologies to reduce inequalities in the determinants of health

Schedule (times are not exact, changed flexibly according to the workshop's progress)

00: 00-00: 15/20 Introduction

Introductory Workshop - Moderator; hello-hello, my name is ... and do ... (about 5 minutes)

Purpose of the PI: audiences outside the research group will help the group achieve better research results.

The project: research process has reached the interpretation phase, key stage, but analytical results without interpretation says nothing - but: no one can know and think about everything, it also applies to scientists.

Demetriq revolves around the question: how does the development of labor market policies affect the chances of different socio-economic groups on the labor market and the risk of exclusion (which is considered to have a connection to the health inequalities between different social groups).

This is where you enter: actors with expertise with respect to the labor market were invited, who can contribute with different views than a social medicine research group has - it is you who are [name of public group] [experts], but not only you, but also

For peer review only

politicians, employers, unions, social insurance office, unemployment agency => to get the "full picture" to broaden the perspective, see more of the analysis results to achieve a better, more reliable interpretation

Use of mp3 player: ok if I start audio recording (capture as much info as possible)

Discussion Rules:

Remember (mp3 player):

 To talk without interruption + to talk one at a time

 To describe the piece of data you refer to with words

 Questions always welcome

Consent:

voluntary – stop at anytime

anonymous - no accounting at the individual level or by name (in the published articles and reports)

confidential - datasets only available to the research team

 Consent in presentation round

Presentation Round: name, position in the organization, consent (identifiable voice on "tape")

Introduction Demetriq - Expert (about 10-15 minutes)

Finish with questions

For peer review only

00: 20 to 00:50: Discussion part I: Employment

<u>1a) presented results (20min): Empty timeline (1) + Employment Charts Sweden (2)</u>

- If you think of the employment rate in Sweden, what events, regulations, developments in international / national level may have contributed to the development of employment (fill in the timeline)? It can be about events or processes regarding policies, but also other areas that you think are relevant (post-it discussion)
- What do you think of when you see the diagram of the employment rate in the various groups (notable parts of the graph) against the background of developments that you mentioned?
- Is it in line with your experience and knowledge?
- In what ways may the development of employment depend on issues you mentioned in the timeline (ideas for explanations, associations)?

<u>1b) presented results (10 min): Employment Charts for SE, DK, NL, UK, compared</u>

- What do you think when you see this European picture where all graphs show a negative trend for the target group?
- What do you think about Sweden's position in the European comparison?

00: 50-01: 10: Discussion part II: poverty risk

<u>Presented results (20 min): Poverty Risk SE, DK, NL, UK</u>

- What do you think of when you see the diagram of the risk of poverty in the different countries (notable parts of the diagram)?
- Is it in line with your experience and knowledge?

For peer review only

• Do you see a connection between poverty and issues you mentioned that you mentioned on the timeline? What then do you think causes the evolution of poverty risk?

01: 10-01: 30 Discussion part III: policy implications

Presented results (20 min): empty logic model

• What regulations and actions are hiding behind the concepts ALMP, flexibility, economic security? (still possible to add something)

• We would like to ask you to fill in the consequences you think that policies affecting economic security, flexibility and active labor market policies may have on employers and employees.

• We have come to this model (show), what do you think? Does it help with carrots / sticks?

01: 30-01: 45: Discussion part IV: flexicurity in Sweden

Presented results (15 min): 4-field diagram with countries DK, NL, UK, without SE

• Position Sweden in the table according to how flexible and secure you feel the Swedish labor market is, and state why you place Sweden where you see it in the table.

• Has there been development over the last 20 years that you can describe and what do you think the future development will look like?

What do these findings say on how to reduce inequalities in health in society? Have we selected the right target group (low edu LLI) and indicators data in that regard?

01: 45-02: 00: End of the Workshop: round up

• What do you take home of what we've talked about today (the most important)? Please summarize briefly.

For peer review only

• What should we take home as a form of research assignments, i.e. questions, focus, and the like for future research from your perspective as representatives of [name of public or stakeholder group]?

For peer review only

BMJ Open

Involving the public in epidemiological public health research: a qualitative study of public and stakeholder involvement in evaluation of a population-wide natural policy experiment

Journal:	*BMJ Open*
Manuscript ID	bmjopen-2017-019805.R2
Article Type:	Research
Date Submitted by the Author:	30-Jan-2018
Complete List of Authors:	Anderson de Cuevas, Rachel; University of Liverpool, Public Health & Policy<br>Nylén, Lotta; Karolinska Institutet, Department of Public Health Sciences, Division of Social Medicine<br>Burström, Bo; Karolinska Institutet, Department of Public Health Sciences, Division of Social Medicine<br>Whitehead, Margaret; University of Liverpool, Public Health and Policy
**Primary Subject Heading**:	Public health
Secondary Subject Heading:	Research methods
Keywords:	social determinants health, health inequalities, public involvement, natural policy experiment, PUBLIC HEALTH, flexicurity

SCHOLARONE™
Manuscripts

For peer review only

Involving the public in epidemiological public health research: a qualitative study of public and stakeholder involvement in evaluation of a population-wide natural policy experiment

Authors:

Rachel Anderson de Cuevas[1], Lotta Nylén[2], Bo Burström[2*], Margaret Whitehead[1]

Affiliated institutions:

[1]Department of Public Health and Policy, University of Liverpool, Liverpool, Merseyside, United Kingdom; [2]Department of Public Health Sciences, Karolinska Institutet, Stockholm, Sweden.

*Corresponding Author: Bo Burström, Professor of Social Medicine, Division of Social Medicine, Dept. of Public Health Sciences(PHS), Karolinska Institutet, K9, Social Medicin, 171 77 Stockholm, Sweden, Email: Bo.Burstrom@ki.se

Keywords: health inequalities; social determinants of health; Europe; public involvement; flexicurity.

Word count: 3995

For peer review only

Abstract

Background: Public involvement in research is considered good practice by European funders; however evidence of its research impact is sparse, particularly in relation to large-scale epidemiological research.

Objectives: To explore what difference public and stakeholder involvement made to the interpretation of findings from an evaluation of a natural policy experiment to influence the wider social determinants of health: 'Flexicurity'.

Setting: Stockholm County, Sweden.

Participants: Members of the public from different occupational groups represented by blue-collar and white-collar trade union representatives. Also, members of three stake-holder groups: the Swedish national employment agency; an employers' association and politicians sitting on a national labour market committee. Total: 17 participants.

Methods: Qualitative study of process and outcomes of public and stakeholder participation in four focussed workshops on the interpretation of initial findings from the Flexicurity evaluation.

Outcome measures: New insights from participants benefitting the interpretation of our research findings or conceptualisation of future research.

Results: Participants sensed more drastic and nuanced change in the Swedish welfare system over recent decades than was evident from our literature reviews and policy analysis. They also elaborated hidden developments in the Swedish labour market which were increasingly leading to "insiders" and "outsiders", with differing experiences and consequences for financial and job security. Their explanation of the differential effects of the various collective agreements for different occupational groups was new and raised

For peer review only

further potential research questions. Their first-hand experience provided new insights into how changes to the social protection system were contributing to the increasing trends in poverty among unemployed people with limiting long-standing illness. The politicians provided further reasoning behind some of the policy changes, and their intended and unintended consequences. These insights fed into subsequent reporting of the Flexicurity evaluation results, as well as the conceptualisation of new research that could be pursued in a future programme.

Strengths and limitations of this study

- To our knowledge, this is the first report of the impact of involving the public and stakeholders in population-wide epidemiological studies, which require interpretation of national quantitative datasets.
- It provides a reasoned solution to the challenge of developing the most appropriate public and stakeholder involvement for quantitative public health research on the wider social determinants of health.
- A limitation is that the public and stakeholders were involved in one discrete phase of the research process, rather than throughout.
- This limitation is being addressed by the continued involvement of participants in the conceptualisation and planning of future research from their ideas coming out of the current study.

For peer review only

Introduction and background

Public and patient involvement in health and social research is regarded by funding bodies and policy makers as an integral part of good practice and democratization of the research process (1). There is growing recognition that lay people have valuable expertise that should inform the research process, as well as having the right to be more actively involved in the design and outputs of research that is about and for them. Increasingly, research funding bodies also require researchers to demonstrate the value of patient and public involvement by evidencing its impact (2).

There is now a substantial body of evidence on good practice in involving patients in research to improve the services that they need (3-5) and a growing literature on involving the wider public in community-based prevention and health promotion research, for example engaging residents of areas in which neighbourhood initiatives are based (6, 7). There are few studies, however, about the involvement of the public in population-wide epidemiological studies, including the evaluation of nation-wide policies to influence the wider social determinants of health (such as employment and poverty). Arguably, one of the main reasons for this dearth of public involvement studies at the population level is the many additional challenges that public health research of this nature throws up. Not least among these challenges is the selection of the most appropriate representatives from amongst “the general public” and the question of where in the process, and in what form, that involvement would be both effective and acceptable to those involved, given the highly technical nature of the statistical analysis of epidemiological datasets.

In this paper, we aim to present the findings of our attempts to tackle some of these challenges, by incorporating public and stakeholder involvement in the evaluation of the

For peer review only

impact on health inequalities of a natural policy experiment in labour market 'flexicurity'. We explain what we did and why, and how that involvement benefitted the interpretation of our research findings and conceptualisation of future research.

'Flexicurity' and the employment of people with a disability or chronic illness

There is a general rise across Europe in the numbers of disabled and chronically ill people of working age that are not in employment. This is a matter of growing concern for governments due to its adverse consequences for public health and potential to exacerbate health inequalities, as well as its effect on increasing social security expenditure (8). Ageing populations and a raised retirement age in several countries mean that the proportion of the working age population leaving the labour force due to ill-health is expected to rise further (8, 9).

In Sweden, the proportion of the population receiving sickness or activity compensation has trebled in the last 30 years (10). The combined cost of sick leave and disability pensions equates to approximately 4% of Swedish GDP and nearly 8% of the working age population receive disability pensions (8). The situation in Denmark and Norway is similar (8 and 10%, respectively), while in the UK, more than 2.6 million people receive incapacity-related benefits, accounting for a quarter of the social security budget and 1.5% of GDP (11).

Being outside the labour market is likely to add to the social and economic exclusion of people with a disability or illness, as income, opportunities to participate fully in society and quality of life decrease (8, 9). People in poor health have lower employment prospects than the general population and these prospects diminish in line with declining socioeconomic position (12, 13). The social gradient in the employment of people with disabilities may serve to widen health inequalities, since the more disadvantaged who already have a higher

For peer review only

prevalence of ill health will be further disadvantaged by the negative effects of unemployment (9).

European countries have undertaken a range of labour market initiatives which may affect the ability of chronically ill and disabled people to get and keep a job (8). Population-wide policies centre around two axes: how flexible the labour market is (the degree of regulation and employment rights) and how much financial security the welfare system offers to disabled people who are out of work. These policies have generated considerable debate about their possible impact on the employment opportunities of chronically ill and disabled people in different socio-economic groups.

Increased flexibility of employment conditions is considered to have contributed to higher levels of job insecurity and to have disproportionately affected those who find themselves in the most vulnerable positions in the labour force (including women, the low skilled and people with disabilities) (14). Since labour market deregulation is considered an essential strategy to redress the economic downturn, 'flexicurity' has been proposed as an alternative; compensating for increased flexibility with provisions of security (10). Flexicurity policies aim to integrate flexible labour market with strong financial security and active labour market policies. (Denmark and the Netherlands are often cited as exemplars of flexicurity in its most developed form). As part of the DEMETRIQ project funded by an FP7 EU grant, we investigated the impact of flexicurity policies on social inequalities in the chances of being in employment for people with and without limiting longstanding illness in European countries. To do this, we exploited, for research purposes, the contrasts in flexibility and financial security found in exemplar countries, which provided the necessary

For peer review only

variation for a natural policy experiment (see for example (15)). The case study in public involvement reported here was conducted as a component of the Swedish country study.

Study design and methods

The qualitative study aimed to explore how public and stakeholder involvement could be incorporated effectively into the evaluation of the health inequalities impact of flexicurity policies and how that affected (if at all) the interpretation of findings and implications for future research.

Study participants: The first question to be resolved is "who is 'the public?' in the context of the evaluation of population-wide employment policies. As the umbrella project was focused on inequalities in health, the EU commissioning brief identified an additional need for "further research on involving population groups with least power and resources, who are intended to be the ultimate beneficiaries of the research" (16). With this proviso and the population level context in mind, we made the following selection decisions:

1. The ultimate beneficiaries of our flexicurity research were people in the most vulnerable positions in the labour market, specifically groups with low skills/low education. Those with low education and disabilities/long-standing illness were doubly disadvantaged.

2. Participants for this explorative qualitative study would be drawn from one European country: Sweden, from where the overall leadership of the flexicuirty evaluation was based, and which was representative of a recognised type of flexibility and security policy system (i.e. low flexibility and high security).

For peer review only

3. We needed to avoid 'tokenism', whereby a few low skilled workers or unemployed people with disabilities were selected at random, without consideration of the nature of the research data they would be engaging with. Following discussions with PPI experts and deep knowledge of the health inequalities literature, we decided that it would be more appropriate to involve trade union representatives from blue-collar and white collar unions as members of the relevant public. They were workers themselves, but also had the added experience of how their fellow workers in different socio-economic circumstances and with different health conditions fared under the various national policies. They were also familiar with considering labour market trends at the population level. In addition, we decided to invite participation from representatives of other relevant public bodies, who could be more accurately termed 'stakeholder groups' rather than 'members of the public'. These stakeholders were members of: the Swedish employers' association (as the Swedish labour market is reliant to a greater extent than elsewhere on collective agreements between trade unions and employers); the Swedish national employment agency (as the public body that implements policies to get unemployed people back to work, and has a special remit for disabled and chronically ill workers); and politicians from the ruling coalition and opposition parties (who sat on a national labour market committee).

Study design and conduct: The study component reported here comprised qualitative research involving members of the public (as defined above) and stakeholder groups in the interpretation of research findings and reflections on future research. Four focused workshops with participants were held using a process developed by Petticrew and

For peer review only - http://bmjopen.bmj.com/site/about/guidelines.xhtml

Whitehead (17, 18). Trade union and stakeholder participants were identified from the research group's existing research users' network, using a snowballing technique. Potential participants were sent invitation letters, an outline of the research project and the objectives of the public involvement activity. Interested participants were given further information about the research and engagement activity through telephone conversations, face-to-face meetings and written correspondence.

The planning, process and outcomes of the focused workshops were recorded and evidenced in parallel to the activity. Preliminary research results were presented to participants in graphical form during focused workshops, and then participants' perspectives on selected research findings elicited.

Two researchers with expertise in qualitative research methods and public engagement, who were independent from the primary study, designed and led the public involvement activity and conducted the analysis. An independent moderator facilitated the workshops.

Description of focused workshop activity

Selected findings were presented at four 2-hour focused workshops held separately with each public and stakeholder group at their workplace during June and August 2014. Two to six participants attended each session, with a total of 17 participants. Findings were presented in diagrammatic form (as described in Box 1). One researcher moderated the discussion and 1 or 2 members of the core research team presented the findings. A standardised topic guide was followed (Appendix 1), with additional questions tailored to suit each group's expertise and interest. Focused workshops were conducted and transcribed in Swedish and selected quotations translated into English.

For peer review only

Box 1: Description of material presented at the focused workshops

Participants were presented with a graph showing trends in Swedish employment rates of people with a limiting longstanding illness (LLI) and healthy people stratified by education level from 1990 to 2011, constructed from the Swedish Survey on Living Conditions and a blank timeline for the same period. Participants were then invited to chart important events or trends during this period on the timeline and to interpret developments in the employment of people with LLI and healthy people in the light of these events. Participants then compared graphs presenting differences in the employment rate of those with and without LLI and with high and low educational levels in Sweden, Denmark, the Netherlands and the United Kingdom for the period 2005 – 2011 from EU SILC data (European Union Statistics of Income and Living Conditions, EUROSTAT). They commented and reflected on the situation in Sweden compared with the other 3 countries. The differential rate of 'poverty risk' (<60 per cent of median income) among non-employed/employed people with LLI and employed healthy people was also presented in graphical form (EU SILC) and participants were asked to interpret changes in poverty risk in light of the key socio-political or economic events they had charted on the timeline. Lastly, participants considered the categorisation of the UK, Netherlands and Denmark in relation to their degree of employment protection and economic security (Flexicurity) in a 2x2 matrix and reflected where to best position Sweden and the trajectory of the country's position over time.

Analytic framework

Full transcripts of the digital recordings of the focused workshops were coded and categorised, employing the principles of content analysis, for the dataset as a whole and

For peer review only - http://bmjopen.bmj.com/site/about/guidelines.xhtml

separately for each workshop (19, 20). Thematic analysis of the moderator's notes and the transcripts drew out key themes from each workshop to form a summary of themes across the workshops and to compare the insights of the 4 different public and stakeholder groups between workshops (21). Transcription and analysis were undertaken alongside the focused workshop activities as part of an iterative process which informed each phase of data collection (22). The Swedish and UK research teams as a whole then reflected on the findings from the focussed workshops and considered how these informed the researchers' original interpretation of their epidemiological data or generated new research questions for future research.

Research ethics

Ethical clearance was not required under Swedish law on ethical review (refer to Lag 2003:460), as the information collected was not considered to constitute sensitive personal information (§3 in the Law). Stakeholders participated as collective members of their employing institution, not as individuals or the subjects of the research. All gave informed consent to being interviewed and for the interviews to be recorded and transcribed.

For peer review only

Results

Participants were all employed women and men aged 40 to 60 years (17 participants). All groups commented on the decline in the employment rate of low educated people with LLI over time and offered a range of explanations for this development (Table 1).

For peer review only

Table 1. Summary of explanations for epidemiological results from participants of focussed workshops.

What explains lower employment rate in target group?	Which policy changes have occurred?	What explains increasing rates of relative poverty among non-employed?	Has Sweden moved to flexicurity?
Globalisation: higher demands on workers, higher education requirements.<br>Economic recessions early 1990s, early 2000 and 2008. (TU, E, EA, P)	Fighting inflation instead of unemployment, to be competitive (TU).<br>De-regulation of many sectors (e.g. transport, construction) (TU)	Increasing market incomes, tax reductions. (P)<br>Stagnant levels of unemployment and social assistance benefits. (P)	Varies between individuals – if you are part of unemployment insurance and collective agreements, you are secure; otherwise not. Labour market not flexible (E).
Changing labour market:<br>New complex technology – increased demand on workers.<br>Reduced demand for unqualified workers (TU, E, EA, P)	Less emphasis on vocational training and life-long learning (TU, EA)	Security has moved to other arenas – collective agreements and individual choices more important (TU, E)	Flexibility for employers has increased. Security has become differentiated – not general. Depends on where you are in the system (TU)
Changing composition of group with low education and LLI – more low educated immigrants with refugee background.<br>(E, EA)	Social security reform in 2008 - reduced duration of sickness absence, restrictions on disability pension (TU, E, EA, P).	Fewer eligible for disability pension, fewer covered by unemployment insurance (EA)	Decreased economic security, not increased flexibility for the employer. Varies between different employers and white/blue collar trade unions, depends on which sector. (EA)
Increasing levels of mental ill health – more difficult to find jobs, lock-in effects. Focus on individual's efforts. (EA)	Reduced economic security to incentivize work (EA)	Reduced economic security to incentivize work (EA)	Politicians divided on the issue – most agree that flexibility is high; economic security is also considered high, but social democrats and green party think security has been lowered (P).
Social insurance reform shifted large groups from social insurance to unemployment (TU, EA)	Security has moved to other arenas – collective agreements and individual choices more important (TU, E)	Increased unemployment insurance fee – lower participation (TU,P)	
Target group less likely to be employed (E)	Extended period for employers to pay sickness benefits (E).	More unemployed rely on social assistance (EA, TU)	

Key: E = employers; P = politicians; TU = trade unions; EA = employment agency.

For peer review only

Explanations for the lower employment rate of low educated people with LLI in Sweden

Participants put forward the economic recessions of the early 1990s, early 2000s and 2008, as explanations for the low employment rate of low educated people with LLI in Sweden: *"That's how it is. We know that in every recession [...] they fall further and further down. They are last in line."* (Trade unionist).

Many considered economic cycles to have had a greater influence on employment rates than national policies. The employer's association and some of the politicians emphasized that the pressures of global competition had led to outsourcing of low-skilled production tasks abroad, placed greater exigencies on workers in employment and removed demand for low-skilled workers with health problems. As one politician put it: *"... we have a labour market which does not have room for these people; as [was the case] ... in the 1990s ..."* (politician)

The 2008 reform of Swedish social insurance limited the time allowed for sickness absence and shifted a large group of people from social insurance to unemployment benefits. This was noted by the employment agency: *"those expelled from the insurance... the first batch ... - [I] think it was 4th January 2010 - ... 14,000 people suddenly came to the employment agency."*

The composition and size of the low educated group with LLI was also seen to have changed. The group had become smaller, as a result of the rise in the educational level of the general population and included a higher proportion of immigrants and people with mental ill-health. This was seen as another explanation for the group's lower employment rate: *"A large proportion of recent immigrants are very low educated or*

For peer review only - http://bmjopen.bmj.com/site/about/guidelines.xhtml

illiterate and of course they have greater difficulties [in securing employment] …" (Employment agency). Another employment agency employee said that it was harder for people with functional disabilities resulting from mental illness to find jobs: *"… it is easier to adjust physically … but if you have ADHD and forget your appointment time …"*

A change in work place attitude towards people with disabilities was also mentioned as a factor contributing to the difficulty for low educated people with LLI to find employment: *"The climate has sort of become tougher"* (*Employment agency)*.

What are the key policy changes in Sweden that have influenced the employment of people with low education and LLI from 1990-2010?

Trade unionists suggested that changes in economic policy underpinned some of the trends in the employment rate of low educated people with activity limitations due to health: *"I believe that the higher structural unemployment that results from prioritizing low inflation over low unemployment enables employers to pick and choose. And then they pick the high educated … rather than the low educated."* Deregulation of many different sectors was also mentioned: *"[The] taxi [industry] in Sweden is the most de-regulated in the world."*

Trade unionists felt that that there had previously been much more emphasis on providing vocational training and continuing education for people with LLI who were not employed, whereas today individuals – particularly blue collar workers – are simply urged to look for and obtain

For peer review only - http://bmjopen.bmj.com/site/about/guidelines.xhtml

a job: *"… you would think our government could step in and make sure these groups get at least an equal chance and support blue collar workers."*

Some participants, including trade union representatives, described how universal social security systems had been eroded over time. Unemployment insurance contributions had increased substantially, resulting in many employees leaving the scheme. Unemployment benefit levels had stagnated or been lowered and social assistance benefits had plateaued.

In parallel with the erosion of universal social security systems, collective agreements between employers and employees had changed, partly to compensate for this phenomenon. As one trade unionist put it:

"Security has moved to other arenas, because, for instance, if you don't increase the unemployment insurance, it has moved to other types of insurance, that's how it is, then parts of the security system have moved."

The "other arenas" included collective agreements. During the discussion, the trade unionists explained that up to the 1990s, Sweden used to have national policies and security systems which would cater for all in the same way, in case of unemployment or redundancy. The active labour market policies (e.g. re-training etc.) benefitted those with lower education. The unemployment insurance was quite generous and adequate (at least for those with low/normal incomes). However, over the years, as the benefit levels were not increased and active labour

For peer review only - http://bmjopen.bmj.com/site/about/guidelines.xhtml

market policies were reduced, the different unions tried instead to supplement with their own insurances, linked to specific collective agreements. The white collar unions with lower unemployment risk could negotiate better terms than blue collar unions – and as a result the differential increased further, and became dependent on the unemployment risk. Those in the most marginal positions on the labour market (and not subscribing to the unemployment insurance) were even worst off.

Employers mentioned another policy influence on people with LLI; an extension to the period of time an employer is responsible for paying sickness benefits for an absent employee, thereby increasing the cost to the employer. This policy change may have been detrimental to the employment chances of persons with LLI. Other reforms to social security regulations, including the aforementioned restriction to the duration of sickness absence and also to eligibility for disability pension, were considered by many participants to be important policy changes aimed at incentivising work: *"It's tougher to be an outsider [to the labour market] today than [it was] 6 years ago … This is partly due to a political desire to strengthen the incentive to work"* (Employment agency).

What explains increasing rates of relative poverty among non-employed persons with limiting longstanding illness in different European countries?

Workshop participants were shown results comparing the prevalence of relative poverty among employed and non-employed people with limiting longstanding illness in Sweden, Denmark, the Netherlands and the United Kingdom, for the years 2005 and 2010, respectively. In

For peer review only - http://bmjopen.bmj.com/site/about/guidelines.xhtml

Sweden, the poverty rate had nearly doubled from 2005 to 2010 albeit from a very low starting point, whereas increases in the other countries, which started with higher poverty rates in 2005, were smaller. Some politicians questioned the validity of the measure "relative poverty" (defined as having an income below 60 percent of the median national income): *"… it describes an increasing income difference in society, but it says nothing about whether poverty has increased. These are two completely different things."*

Other participants attributed the increasing rates of relative poverty directly to national policies: *"… it has been a deliberate policy … to increase the differences in income according to whether you work or do not work, … so you notice the change if you start working"* (Employment agency).

The social insurance reform of 2008 had excluded many non-employed people with LLI from sickness benefit, yet many were not on the unemployment register. The issue was how they made their living: *"… they are somehow supported by relatives … The group with mental problems … seek social assistance more than others"* (Employment agency).

The increase in unemployment insurance contributions (due to a change in government policy in 2007) led to many employees leaving the insurance and was also put forward as an explanation for rising rates of relative poverty among this population group: *"… there are many unemployed people who are not part of the unemployment insurance scheme at all, and who don't get anything …"* (Employment agency).

For peer review only - http://bmjopen.bmj.com/site/about/guidelines.xhtml

Has Sweden moved to flexicurity?

Workshop participants were asked whether they considered that the policy changes that had taken place from 1990 to 2010 had moved Sweden to qualify as a 'flexicure' country and were asked to place Sweden in a matrix of high/low flexibility and high/low economic security (Figure 1). (Denmark, the Netherlands and the UK were positioned by the researchers prior to the workshops in line with the evidence from the policy literature). Opinions were divided. One trade unionist commented: *"The whole of [Swedish] working life has slipped towards the UK [position, with high flexibility/deregulation and low economic security] …"* Another trade unionist also thought that flexibility had increased: *"In Sweden, we have perhaps the highest flexibility out of many countries, to fire people … It is incredibly liberal … We belong to high flexibility."*.

Employers, on the other hand, considered that flexibility was low, but that the degree of economic security differed according to whether or not an employee was part of unemployment insurance and covered by collective agreements. For those who did not belong to these schemes, security was lower than before: *"… the universal welfare system has been hollowed out since the 1990s"* (Employer).

Trade unionists and employers referred to a two-tier system, in which the situation for 'insiders' to the labour market - those who have had a job for some time, who are part of the unemployment insurance scheme and are covered by collective agreements - was much better than for those outside these arrangements. Most politicians commented that Sweden had high flexibility and high security, having moved from lower

For peer review only - http://bmjopen.bmj.com/site/about/guidelines.xhtml

flexibility and even higher economic security. One politician spoke of: *"… a divided labour market … There are those who have security and those who do not …"*

For peer review only

For peer review only - http://bmjopen.bmj.com/site/about/guidelines.xhtml

Impact of this involvement on researchers' interpretation of findings

Subsequent to the focused workshops with public and stakeholder representatives, the wider research team comprising Swedish and UK members held reflective sessions to consider if, and how, the involvement activity had influenced their initial interpretation of findings or highlighted gaps in the evidence base.

Four main insights were identified, triggered by the public and stakeholder involvement, which deepened our understanding of our findings and influenced subsequent publications. First was the extent to which people experiencing the labour market and welfare reforms first hand (at 'the coal face' so to speak) sensed more drastic and nuanced change in the Swedish welfare system over the past two decades than was evident from the academic literature reviews and policy analyses. The exercise resulting in figure1 highlighted this point visually.

Second was the introduction of the concept of "insiders" and "outsiders" in the labour market, emerging from the workshops, particularly from discussions among trade unionists and employers. This highlighted the differing experiences and consequences for employees depending not just on SES or simple blue-collar/white collar dychotomy, but also on the length of time employees had had a job and whether or not they were part of the whole infrastructure which provided financial and job security. This concept fed into the generation of hypotheses to be tested in secondary data analysis, the results of which we subsequently published in a peer-reviewed paper (15).

Third was the possibility of differential effect of collective agreements for different occupational groups. It was the deep understanding by unions and employers of how

For peer review only - http://bmjopen.bmj.com/site/about/guidelines.xhtml

collective agreements had operated in practice over the years that offered the researchers fresh insights into how groups that were already disadvantaged could be hardest hit by poorer, or no, collective agreements. This led to the development of a research proposal on the role of collective agreements in contributing to differential impacts of labour market policies, which was submitted by the research team to a Swedish national research funder: FORTE.

Fourth, triangulation of perspectives from the different public and stakeholder groups gave a much fuller picture of what was happening as a result of the interactions of people with the system. The trade unions and employers raised issues about the consequences of collective agreements as detailed above. The employment agency described from first-hand experience the impact of changing policies on the lives and finances of unemployed people, particularly those who were "outsiders". The politicians understood, from their own experience of legislating the stated and real reasons for changes in policies and the intended and unintended consequences.

Discussion

This is a rare example of a study exploring what difference public and stakeholder involvement makes to an evaluation of a natural policy experiment to influence the wider social determinants of health. Furthermore, it was conducted in a European country outside the UK, where the concept of lay involvement is less developed. To our knowledge, this is also the first report of involving the public and stakeholders in population-wide epidemiological studies, which require interpretation of national quantitative datasets (4).

For peer review only - http://bmjopen.bmj.com/site/about/guidelines.xhtml

Reservations have been expressed elsewhere about the difficulties of involving the public in research that is highly technical (23). Added to that was the population-wide nature of the policies being evaluated. Our decisions on *who* to involve and *how* were therefore critical to the whole research endeavour reported here. Our final selection of trade union representatives from blue-collar and white-collar unions as the most appropriate members of the relevant public, coupled with the decision to involve stakeholders from the employers' organisation, public employment office and politicians serving on employment policy committees, proved both feasible and productive. Participants in the focussed workshops demonstrated a high level of lay expertise and a capacity to engage with the intricacies of policy developments, changes to the economy, the country's demographic profile and global market forces, from both a practical and theoretical perspective. They expressed appreciation of the way the study findings were presented to them for comment – as "*big trends over time*" – rather than facing them, for example, with the technicalities of the epidemiological analysis. There is continuing debate about how to facilitate lay involvement in research and at what point would be most appropriate (24-26). The focussed workshops reported here provide an example of what helped public and stakeholder involvement in the context of an evaluation of a population-wide natural policy experiment.

The study concluded that this public and stakeholder involvement benefited the interpretation of the research findings and conceptualisation of future research in a number of ways. Participants sensed more drastic and nuanced change in the Swedish welfare system over recent decades than was evident from our literature reviews and policy analysis. They also elaborated hidden developments in the Swedish labour market which

For peer review only - http://bmjopen.bmj.com/site/about/guidelines.xhtml

were increasingly leading to "insiders" and "outsiders", with differing experiences and consequences for financial and job security. The explanation by the trade unionists and employers of the differential effects of the various collective agreements for different occupational groups was new and had not been a focus in the studies performed by the researchers. The first-hand experience of the employment agency provided new insights into how changes to the social protection system were contributing to the increasing trends in poverty among unemployed people with limiting long-standing illness. The politicians provided further reasoning behind some of the policy changes, and their intended and unintended consequences. These insights fed into our subsequent reporting of the results of the Flexicurity evaluation (15, 27, 28), as well as the conceptualisation of new research that could be pursued in a future programme (see below).

Limitations

The main limitation was that public and stakeholder involvement was limited to one discrete phase of the research process: the interpretation of initial findings. There is some evidence to suggest that involving the public throughout the research process (as well as long-term involvement and continuity of membership beyond a single research project) enhances impact (4, 29, 30). While we judged involvement in this discrete phase to be the most practical and logical starting point faced with the specific population-wide policy evaluation of flexicurity, it became clear during the focussed workshops that this could also be a way of building a continued relationship for future research. The research ideas coming out of the workshops and the willingness on the part of participants to be involved in a future programme, laid the groundwork for involvement in future research from the very beginning, including the conceptualisation of the research questions.

For peer review only - http://bmjopen.bmj.com/site/about/guidelines.xhtml

A higher-level limitation is that research on public and stakeholder involvement is inherently context-specific, as the impact of involvement is likely to depend on many factors, including the topic being studied, the public groups being represented, the perspective of the researchers, and the extent of involvement. Producing generalizable evidence in the area of public involvement is inherently difficult. This study was conducted in the context of Sweden and its labour market and social protection system. Mindful of this, we tried to tailor the public and stakeholder involvement initiatives to take account of the country context (Sweden), the topic (policies around Flexicurity) and the nature of the research in which involvement is being encouraged (population-wide epidemiological studies). In this type of research, understanding and taking account of the context is essential if the findings are to be of value to research users in Sweden and elsewhere.

Input into the conceptualization stage of future research

The public and stakeholder involvement, although only at the interpretation of findings stage for the current research, gave rise to new research questions to address in a future research programme. Could it therefore be a way of introducing public involvement right from the conceptualization stage in future population-wide epidemiological studies of this nature? Ideas for future research that came out of the public and stakeholder involvement included the following.

First was the question of how the changing composition of, and the increasing prevalence of neuropsychiatric diseases in, the group of low educated people with LLI might affect the employment chances of the group. This is an interesting issue to pursue in view of the increasing demand for social skills on the labour market, also in lower skilled jobs.

For peer review only - http://bmjopen.bmj.com/site/about/guidelines.xhtml

Another idea for future studies came from the unionists' statement of differential conditions associated with different collective agreements, that "security has moved to other arenas", i.e. from the national level to the level of specific agreements negotiated by individual unions. Here, detailed assessment of conditions pertaining to different groups of manual workers, compared to conditions among higher non-manual employees, could help identify differential impacts and trajectories of different groups in the event of unemployment. This idea has been worked up, jointly with participants, into a research proposal, which was submitted to a Swedish national funder, FORTE.

Thirdly, the employment agency offered the use of their more detailed data to study the employment and economic situation of specific groups, such as people with psychological illnesses, whose employment prospects and economic situation may be poorer than among those with physical illnesses, not least following recent restrictions in social insurance policies.

Hence, early contacts with the public and with stakeholders, who day to day face the concrete impact of new policies or other changes on the labour market, may be very pertinent to specifying research questions addressing the impact of such real life changes. Increased involvement and dialogue with the public and with stakeholders in the identification and framing of specific research questions may therefore contribute both to increased scientific rigour, as well as societal relevance of policy oriented research.

For peer review only - http://bmjopen.bmj.com/site/about/guidelines.xhtml

Conclusion

This study shows that it is possible to incorporate public and stakeholder involvement in a meaningful way in epidemiological public health research. The insights and reflections the public and stakeholders offered added depth and subtlety to the researchers' interpretation of the research findings and highlighted the issues perceived to be of most public relevance. In addition, participants raised questions that could not be answered by the current study, which stimulated ideas for future research and contributed to specifying highly relevant research questions. Early engagement with the public and with stakeholders may therefore contribute to improve both the scientific rigour and the relevance of policy oriented research.

For peer review only - http://bmjopen.bmj.com/site/about/guidelines.xhtml

Contributorship statement:

MMW and BB conceived the research study. LN and RMAdC designed and led primary data collection and analysed the data. RMAdC and BB wrote the first draft and together with MMW and LN interpreted the data. All authors contributed to the final draft.

Competing interests:

The authors have no conflicts of interest to declare.

Funding:

This research was funded by an European Commission FP7 grant DEMETRIQ (developing methodologies to reduce inequalities in the determinants of health) (Grant Reference: 278511), http://www.demetriq.eu/. The funders had no role in study design, data collection and analysis, decision to publish, or preparation of the manuscript. The authors of this paper alone are responsible for the views expressed in this publication which do not necessarily represent the decisions or policies of the EU or their institutions.

Data sharing statement:

Transcripts of the focussed discussions, field notes and observations are in Swedish. To protect the anonymity of the public and stakeholder participants, we cannot share the primary qualitative data with third parties.

For peer review only - http://bmjopen.bmj.com/site/about/guidelines.xhtml

References

1. Boote J, Baird W, Beecroft C. Public involvement at the design stage of primary health research: a narrative review of case examples. Health policy (Amsterdam, Netherlands). 2010;95(1):10-23.
2. Snape D, Kirkham J, Britten N, Froggatt K, Gradinger F, Lobban F, et al. Exploring perceived barriers, drivers, impacts and the need for evaluation of public involvement in health and social care research: a modified Delphi study. BMJ open. 2014;4(6):e004943.
3. Brett J, Staniszewska S, Mockford C, Herron-Marx S, Hughes J, Tysall C, et al. Mapping the impact of patient and public involvement on health and social care research: a systematic review. Health expectations. 2012;17(5):637-50.
4. Staley K. Exploring impact: Public involvement in NHS, public health and social care research. Eastleigh: INVOLVE, 2009.
5. Ocloo J, Matthews R. From tokenism to empowerment: progressing patient and public involvement in healthcare improvement. BMJ quality & safety. 2016;25(8):626-32.
6. O'Mara-Eves A, Brunton G, McDaid D, Oliver S, Kavanagh J, Jamal F, et al. Community engagement to reduce inequalities in health: a systematic review, meta-analysis and economic analysis. Public Health Res. 2013;1(4).
7. GoWell. GoWell Community Engagement: GoWell Glasgow Community Health and Wellbeing: Research and Learning Programme 2018 [updated 2018. Available from: http://www.gowellonline.com/about/components/community_engagement.
8. Whitehead M. Disability and employment: lessons from natural policy experiments. European journal of public health. 2010;20(4):371-3.
9. Clayton S, Barr B, Nylen L, Burstrom B, Thielen K, Diderichsen F, et al. Effectiveness of return-to-work interventions for disabled people: a systematic review of government initiatives focused on changing the behaviour of employers. European journal of public health. 2012;22(3):434-9.
10. Effective return to work. Reforms. Stockholm: Government Offices of Sweden, 2008.
11. Clayton S, Bambra C, Gosling R, Povall S, Misso K, Whitehead M. Assembling the evidence jigsaw: insights from a systematic review of UK studies of individual-focused return to work initiatives for disabled and long-term ill people. BMC public health. 2011;11:170.
12. Burstrom B, Holland P, Diderichsen F, Whitehead M. Winners and losers in flexible labor markets: the fate of women with chronic illness in contrasting policy environments--Sweden and Britain. Int J Health Serv. 2003;33(2):199-217.
13. Burstrom B, Whitehead M, Lindholm C, Diderichsen F. Inequality in the social consequences of illness: how well do people with long-term illness fare in the British and Swedish labor markets? Int J Health Serv. 2000;30(3):435-51.
14. Afzal Z, Muntaner C, Chung H, Mahmood Q, Ng E, Schrecker T. Complementarities or contradictions? Scoping the health dimensions of "flexicurity" labor market policies. Int J Health Serv. 2013;43(3):473-82.
15. McAllister A, Nylen L, Backhans M, Boye K, Thielen K, Whitehead M, et al. Do 'Flexicurity' Policies Work for People With Low Education and Health Problems? A Comparison of Labour Market Policies and Employment Rates in Denmark, The Netherlands, Sweden, and the United Kingdom 1990-2010. Int J Health Serv. 2015;45(4):679-705.
16. EC. FP7-HEALTH-2011-two-stage: Cooperation programme: European Commission; 2010 [updated 20-07-2010. Available from: https://ec.europa.eu/research/participants/portal/desktop/en/opportunities/fp7/calls/fp7-health-2011-two-stage.html.
17. Whitehead M, Petticrew M, Graham H, Macintyre SJ, Bambra C, Egan M. Evidence for public health policy on inequalities: 2: assembling the evidence jigsaw. Journal of epidemiology and community health. 2004;58(10):817-21.

For peer review only - http://bmjopen.bmj.com/site/about/guidelines.xhtml

18. Petticrew M, Whitehead M, Macintyre SJ, Graham H, Egan M. Evidence for public health policy on inequalities: 1: the reality according to policymakers. Journal of epidemiology and community health. 2004;58(10):811-6.
19. Robson C. Real World Research. 2nd edition ed. Oxford: Blackwell; 2002.
20. Graneheim UH, Lundman B. Qualitative content analysis in nursing research: concept, procedures and measures to achieve trustworthiness. Nurse Education Today. 2004;24(105-112).
21. Braun V, Clarke V. Using thematic analysis in psychology. Qualitative research in psychology. 2006;3(2):77-101.
22. Ritchie J, Lewis J, editors. Qualitative research practice: a guide for social science students and researchers. London: Sage Publications Limited; 2003.
23. Barber R, Boote JD, Parry GD, Cooper CL, Yeeles P, Cook S. Can the impact of public involvement on research be evaluated? A mixed methods study. Health expectations. 2012;15(3):229-41.
24. The Public Involvement Impact Assessment Framework Guidance. . Universities of Lancaster, Liverpool and Exeter, 2014.
25. PiiAF. Public involvement impact Assessment Framework (PiiAF) Resources: Draft standards for good practice in public involvement in research. MRC, 2014.
26. Gradinger F, Britten N, Wyatt K, Froggatt K, Gibson A, Jacoby A, et al. Values associated with public involvement in health and social care research: a narrative review. Health Expectations. 2013.
27. Backhans MC, Mosedale S, Bruce D, Whitehead M, Burstrom B. What is the impact of flexicurity on the chances of entry into employment for people with low education and activity limitations due to health problems? A comparison of 21 European countries using Qualitative Comparative Analysis (QCA). BMC public health. 2016;16(1):842.
28. Burström B, et al. Demetriq Project Report to the European Union. 2015.
29. Staniszewska S. Patient and public involvement in health services and health research: A brief overview of evidence, policy and activity. Journal of Research in Nursing. 2009;14(4):295-8.
30. PiiAF. Database – Methods and tools to assess impacts: Signposting resource to published case examples of methods and tools. PiiAF, 2013 Contract No.: 1st May 2014.

Fig 1: Matrix showing the different categorizations of Sweden's degree of Flexicurity by the 4 public groups

For peer review only - http://bmjopen.bmj.com/site/about/guidelines.xhtml

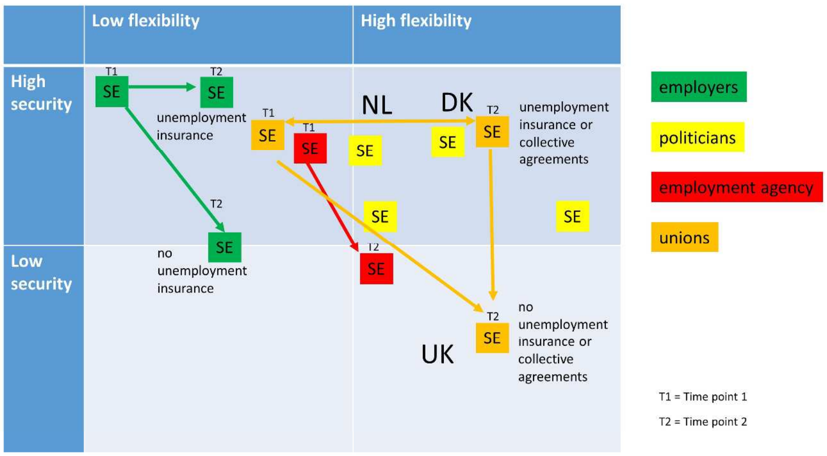

Fig 1: Matrix showing the different categorizations of Sweden's degree of Flexicurity by the 4 public groups

338x190mm (300 x 300 DPI)

S1 Appendix 1: Focused Workshop standard topic guide

Public

WS-leader Expert (introduce Demetriq + results), Moderator (questions, discussion)

Dual audio recording (mp3 + smartphone)

Demetriq = Developing methodologies to reduce inequalities in the determinants of health

For peer review only

Schedule (times are not exact, changed flexibly according to the workshop's progress)

00: 00-00: 15/20 Introduction

Introductory Workshop - Moderator; hello-hello, my name is ... and do ... (about 5 minutes)

Purpose of the PI: audiences outside the research group will help the group achieve better research results.

The project: research process has reached the interpretation phase, key stage, but analytical results without interpretation says nothing - but: no one can know and think about everything, it also applies to scientists.

Demetriq revolves around the question: how does the development of labor market policies affect the chances of different socio-economic groups on the labor market and the risk of exclusion (which is considered to have a connection to the health inequalities between different social groups).

This is where you enter: actors with expertise with respect to the labor market were invited, who can contribute with different views than a social medicine research group has - it is you who are [name of public group] [experts], but not only you, but also

politicians, employers, unions, social insurance office, unemployment agency => to get the "full picture" to broaden the perspective, see more of the analysis results to achieve a better, more reliable interpretation

Use of mp3 player: ok if I start audio recording (capture as much info as possible)

Discussion Rules:

Remember (mp3 player):

 To talk without interruption + to talk one at a time

 To describe the piece of data you refer to with words

 Questions always welcome

Consent:

voluntary – stop at anytime

anonymous - no accounting at the individual level or by name (in the published articles and reports)

confidential - datasets only available to the research team

 Consent in presentation round

Presentation Round: name, position in the organization, consent (identifiable voice on "tape")

Introduction Demetriq - Expert (about 10-15 minutes)

Finish with questions

For peer review only

00: 20 to 00:50: Discussion part I: Employment

1a) presented results (20min): Empty timeline (1) + Employment Charts Sweden (2)

- If you think of the employment rate in Sweden, what events, regulations, developments in international / national level may have contributed to the development of employment (fill in the timeline)? It can be about events or processes regarding policies, but also other areas that you think are relevant (post-it discussion)
- What do you think of when you see the diagram of the employment rate in the various groups (notable parts of the graph) against the background of developments that you mentioned?
- Is it in line with your experience and knowledge?
- In what ways may the development of employment depend on issues you mentioned in the timeline (ideas for explanations, associations)?

1b) presented results (10 min): Employment Charts for SE, DK, NL, UK, compared

- What do you think when you see this European picture where all graphs show a negative trend for the target group?
- What do you think about Sweden's position in the European comparison?

00: 50-01: 10: Discussion part II: poverty risk

Presented results (20 min): Poverty Risk SE, DK, NL, UK

- What do you think of when you see the diagram of the risk of poverty in the different countries (notable parts of the diagram)?
- Is it in line with your experience and knowledge?

For peer review only

• Do you see a connection between poverty and issues you mentioned that you mentioned on the timeline? What then do you think causes the evolution of poverty risk?

01: 10-01: 30 Discussion part III: policy implications

Presented results (20 min): empty logic model

• What regulations and actions are hiding behind the concepts ALMP, flexibility, economic security? (still possible to add something)

• We would like to ask you to fill in the consequences you think that policies affecting economic security, flexibility and active labor market policies may have on employers and employees.

• We have come to this model (show), what do you think? Does it help with carrots / sticks?

01: 30-01: 45: Discussion part IV: flexicurity in Sweden

Presented results (15 min): 4-field diagram with countries DK, NL, UK, without SE

• Position Sweden in the table according to how flexible and secure you feel the Swedish labor market is, and state why you place Sweden where you see it in the table.

• Has there been development over the last 20 years that you can describe and what do you think the future development will look like?

What do these findings say on how to reduce inequalities in health in society? Have we selected the right target group (low edu LLI) and indicators data in that regard?

01: 45-02: 00: End of the Workshop: round up

• What do you take home of what we've talked about today (the most important)? Please summarize briefly.

For peer review only

• What should we take home as a form of research assignments, i.e. questions, focus, and the like for future research from your perspective as representatives of [name of public or stakeholder group]?

For peer review only
